# The Ethnobotanical Heritage of *Olea europaea* L. in Italy: Continuity of a Millenary Mediterranean Tradition

**DOI:** 10.3390/plants15142221

**Published:** 2026-07-21

**Authors:** Rosalucia Mazzei, Luca Lombardo, Samanta Zelasco, Marianna Rizzo, Giuseppe Tagarelli

**Affiliations:** 1 CNR ISMed—Institute for Mediterranean Studies, Via Cardinale Guglielmo Sanfelice, 8, 80134 Naples, Italy; rosalucia.mazzei@cnr.it; 2 CREA OFA—Centro di Ricerca Olivicoltura, Frutticoltura e Agrumicoltura, via Settimio Severo, 83, 87036 Rende, Italy; samanta.zelasco@crea.gov.it (S.Z.); marianna.rizzo@crea.gov.it (M.R.)

**Keywords:** *Olea europaea* L., ethnobotany, Mediterranean shared roots

## Abstract

The olive tree (*Olea europaea* L.) is a quintessential symbol of the Mediterranean basin, representing a profound intersection of ecological adaptation, agricultural evolution, and cultural identity. Italy, where the olive has shaped dietary habits, traditional medicine, folklore, and landscape from prehistory to the present, serves as a primary macro-region for studying the co-evolution of human societies and this iconic plant. In this vein, this paper examines the ethnobotanical trajectory of *O. europaea* on the Italian peninsula through an analysis of traditional knowledge concerning its medicinal and ritual uses, reported in ethnographic literature dating from the second half of the 19th century to the first half of the 20th century. In addition, we conducted a review of Italian ethnobotanical fieldwork published between 2000 and 2025. Accordingly, olive leaves (used as antihypertensives, febrifuges, hypoglycemic and apotropaic agents), bark and roots (to treat various pathologies) and oil (in rituals for spiritual healing and against evil eye, as an emollient for dermatological pathological and cosmetical conditions and as a vehicle for infusing other therapeutic herbs) have found a central place in the Italian popular pharmacopeia and magic-religious practices. The roots of these traditions have been contextualized within a wider Mediterranean oleo-culture rising from the second millennium BCE. By identifying knowledge gaps, documenting regional ethnobotanical diversity, and connecting historical and contemporary evidence, this work provides a novel framework for future comparative ethnobotanical research. Eventually, a discussion on how contemporary Italy is reframing this botanical legacy through the lenses of agrobiodiversity conservation and the safeguarding of historical rural landscapes against modern threats such as climate change and phytosanitary crises is presented.

## 1. Introduction

Recent ethnobotanical field studies have documented the traditional uses of *O. europaea* across numerous Mediterranean countries, with particularly rich evidence from North Africa and the Levant [1]. In contrast, despite Italy representing one of the principal historical centers of olive cultivation and domestication in the Mediterranean, a comprehensive synthesis of its ethnobotanical heritage is still lacking.

The olive tree (*Olea europaea* L. subsp. *europaea*) has a rich cultural and historical legacy in Italy, where it has been cultivated for thousands of years, starting from the proto-forms of wild-olive growing in Sicily, Calabria and Apulia dating to the early Bronze Age (21st–18th century BCE) [2,3,4,5] and evolving through the technical advancement (from the 15th century BCE) introduced by Aegean and Levantine (Phoenicians) peoples [6], and the Roman agronomic standardization. Throughout this period, the olive acquired a primary role in dietary, cosmetic, and medicinal practices, as well in ritualistic functions because of its profound symbolic meaning representing peace, fruitfulness, strength, and purification. Moreover, the olive’s healing properties were frequently mentioned in the context of both practical knowledge and mystical beliefs, often blurring the line between empirical science and magic.

The present study aims to provide a comprehensive overview of the contemporary ethnobotanical heritage of *O. europaea* in Italy. To achieve this, we integrated ethnobotanical data collected through fieldwork conducted between 2000 and 2025 with ethnographic sources spanning from the second half of the nineteenth century to the onset of Italy’s economic boom in the 1950s. This integrated approach enables us to reconstruct the continuity of olive-related traditional knowledge, trace its roots to a shared millenary Mediterranean olive culture, and evaluate its persistence within contemporary Italian folk practices despite the profound socio-cultural changes of recent decades.

### 1.1. The Shared Mediterranean Roots of the Ethnobotanical Use of O. europaea

The olive tree has occupied a leading role in the socio-cultural development of the peoples of the Mediterranean, where it shaped a common oleo-culture based on its ritualistic and pharmacological use since the Bronze Age [7].

#### 1.1.1. Therapeutic Applications

Olive oil’s medical applications became explicitly documented in the Egyptian Medical Papyri. In the Ebers Papyrus (c. 1550–1530 BCE), a comprehensive treatise on Egyptian medicine, olive oil appears as a foundational ingredient in ointments and remedies for diverse pathologies [8]. Shortly after, the Hearst Medical Papyrus (c. 1500 BCE) incorporates olive oil into the preparation of decoctions and medicinal adjurations against the Asiatic disease [9]. In the 2600-year-old Assyrian cuneiform Nineveh Medical Encyclopaedia, found on clay tablets in the library of the king Ashurbanipal, olive oil and olive leaves are among the ingredients for the treatments of fever and cranial (https://www.britishmuseum.org/collection/object/W_K-2354; accessed on 2 June 2026) or neck (https://www.britishmuseum.org/collection/object/W_Rm-116 accessed on 2 June 2026) injuries.

In the Greek world, Hippocrates (460-377 BCE) recommended cataplasm of olive oil and boiled olive leaves to heal ulcers (*De Ulceribus*) and oiling the wrestler’s body to warm it and avoid injuries in winter (*De diaeta*), but throughout the *Corpus Hippocraticum*, olive oil is a supporting element in various therapies for the treatment of ailments ranging from broken bones to hemorrhoids, fistulas, dry cholera, hydrops and polyarthritis. In Pindar’s *Pythian Odes* (5th century BCE) there is a reference to the employment of drugs mixed with olive oil for the treatment of severe pain; meanwhile from Plato we know that according to the 5th century BCE philosopher Protagoras (*Protagoras* 334b, c) olive oil is particularly harmful for all plants and for animals’ hair, and while it has beneficial effects for the outside of the human body (including hair) it has equally negative ones for the inside, meaning doctors did not prescribe oil for the sick. Moreover, in Plato’s short dialogue *Menexenus* (338a), (the birth of) olive oil is saluted as the relief of pain.

Several therapeutic indications (as sedative, laxative, anti-inflammatory and pain-protective) of olive oil are reported in the earliest Greek Medical Papyri [10] of the Graeco-Roman and Byzantine Egypt, dated from the 3rd/2nd century BCE to the 7th century CE.

During the Imperial Roman period, olive growing experienced its greatest expansion in antiquity, so it is not surprising that Latin or Romanized authors dedicated ample space to the virtues of the olive tree and its derivative products.

In Pliny’s *Naturalis Historia* (I century CE), from chapter 34 to 39 of book XXIII, a total of 74 remedies from olive leaves (23 remedies), blossoms (4), drupes (7), *amurca* (21), *omphacium,* the oil from greener olives (3), and wild-olive leaves (16) are reported. Their use through topical application, chewing or ingestion is recommended for a wide variety of health problems ranging from headache to digestive system (including mouth) disorders, eye problems, excessive menstrual flow and especially skin diseases. It is however in the XXX book that Pliny, with a certain skepticism and some irony, offers us a glimpse into the popular gullibility of his time, describing olive oil as the main vector of the improbable curative concoctions proposed by Magicians/Magi (Table S1).

Aulus Cornelius Celsus (I century CE) believed (*De Medicina*) that olive oil was needed for curing eye problems, sunburns, headache, ulcers, fevers and gastric disturbs, whereas olive leaves boiled in wine helped with penis swelling. Olive oil as a skin treatment, pain healer and matrix of other medicinal oils is found in Dioscorides’ *De materia medica* (40–90 CE), in the works of Rufus of Ephesus (I–II century CE), Quintus Serenus Sammonicus (*Medicinalis* XL, 9; III century CE) recommended olive oil use against holy fire (*ignis sacer*). Soranus of Ephesus (II century CE) in the opera *Gynaecia* considered the application of old olive oil as an aid to prevent conception, partially in agreement with Aristotle (*Historia Animalium* VII, 3) asserting that an ointment based on lead or frankincense commingled with olive oil had to be used. Moreover, olive-based treatments were found in Galen (II–III century CE), in the *Collectiones medicae* of Oribasius (IV century CE) and in *Libri Medicinales by* Aetius of Amida (V century CE). Finally, Caelius Aurelianus (V century CE) by translating Soranus’ work (*De morbis acutis et chronicis*) attested that olive oil was employed as a clyster or a vomitive to deworm the human body [11].

With the re-expansion of olive growing in the high Middle Ages, interest in its health properties also reawakened. Medicinal uses of olive oil are found in the *Qānūn fī l-ṭibb* (*The canon of medicine*) of Avicenna (980–1037 CE), in the collection of *Tacuinua Sanitatis* based on the work of the X century physician Ibn Butlan, in *Saydanah fi al-Tibb* (*Book of Pharmacy in Medicine*) by Ahmad al-Biruni (973–1048 AD), in *Physica* and *Causae et Curae* by the XII century German abbess and mystic Hildegard von Bingen (who, however, considered the oil unsuitable for culinary use), and in the teachings of the *Schola Medica Salernitana* [12], the first Western medical school, born between the X and XI century CE, explicated in the book *Regimen Sanitatis Salernitanum*. The health properties of olive oil, especially with topical action, were also recognized in late Middle Ages in writings such as *Commentarii in libros sex Pedacii Dioscoridis Anazarbei De Medica Materia* (1459) by Pierandrea Mattioli and *Opus Pandectarum Medicinae* (1474) by Mattheus Sylvaticus.

#### 1.1.2. Veterinary Uses

For Aristotle (*Historia Animalium* VIII, 26) a sip of olive oil is helpful against iron objects in elephants’ internal organs, though “*some elephants like olive oil*, *and others do not*”, but if they happen to swallow a chameleon, they eat wild olives as a remedy (Pliny, *Nat. Hist.* VIII, 41). Olive oil was even used in the treatment of animal diseases as described in Cato’s *De re rustica*, in Pelagonio’s *Ars veterinaria* (IV century CE), in Palladio’s *Opus agriculturae* in the compendium *Mulomedicina Chironis* (IV century CE) and by derivation in the *Mulomedicine* of Vegezio (IV–V century CE).

#### 1.1.3. Religious Practices

Rubbing the body with “holy” olive oil as a means of conferring both physical and spiritual benefits has its origins in the ancient practice of anointing. The earliest written evidence of olive oil’s ritual significance emerges in the 25th century BCE within the clay tablets of Ebla (Syria). Here, references to the *i-giš sag* ritual has been interpreted as a purification rite through the anointing of the head with olive oil (*i-giš*) during solemn occasions such as funeral and celebratory [13]. By the 13th century BCE, the practice of anointing became deeply embedded in religious literature. This is paradigmatically evident in the Old Testament, where the word “anoint” and its derivatives appear over 130 times, with particular reference to olive oil. The healing sacredness through anointing is also found in the Christian tradition, whereas the evangelist Mark (6:13) stated that the apostles of Jesus, by continuing the work of their Master, “anointed with oil many who were sick and cured them”. Following this belief, in the *Roman Ritual*, a liturgical book of the Catholic Church, the use of blessed oil is foreseen for the cure of and protection from ailments. This has a parallel in the Islamic world with the *ruqyah* (the spiritual healing through reciting prayers or Qur’anic verses) oil, directly deriving from the medieval Prophetic medicine. Ruqyah, was and still is used for conditions like the evil eye, snake bites, scorpion stings and mental disorders. While some hadiths contradict each other about ruqyah, its practice involves reciting specific words, sometimes over materials like olive oil, water, or dust, to treat spiritual and physical issues, including possession by jinn (evil spirits) [14].

In the Punic tradition, olive oil is an important element in the Marseille and Carthage Tariff (5th–3rd century BCE) which reported the prices and descriptions of the selectable purification rituals (with or without animal sacrifices) carried out by priests. In particular, the Marseille punic inscription KAI/I, p.1, ins. #69, line 12 [15], as well as the Carthaginian limestone plaque P74 [16] refer to the “sacrifice of oil” (*ZBH ŠMN*), the ritual, alternative to the sacrifice in blood, in which oil is shed as a redemption/entrustment offering to a deity. A sacrifice of oil was made by Jacob in the Bible (Gen. 28:18; 6th–5th century BCE).

#### 1.1.4. Magical Practices

The Hittites used olive branches in birth rituals [17], and olive oil for the 15th century BCE magical Ammihatna and Maštigga rituals [18] against impurity and domestic quarrel.

An obscure relationship binds the eye of Horus (intended in this case as a celestial food for the blessed) and the olive tree in both the *Unas* texts [19] and the *Egyptian Book of the Dead* [20].

Olive fruits, oil and branches were recurring “ingredients” in the Greek Magical Papyri (PGM), from the 1st century BCE to 4th century CE Egypt collecting rituals for divination, exorcisms, reaching invisibility, necromancy, etc. [21] in the form of magic potions or amulets, with olive wood also present in a Demotic Magical Papyrus [22]. In particular, PGM IV (lines 222–234) describes the employment of green olive oil for lecanomancy, the divinatory practice by interpreting the shapes and movements generated by liquids poured into a dish (λεκάνη -lekắnē-), which, in turn, has a much older predecessor in the two discrete fragments (tablets KUB 37.198 and KUB 34.5) of a single Babylonian cuneiform text (c.a. 2000–1600 BCE) detailing “oil omens” [23]; however, it is likely that the oil referred to in this case was sesame oil. These examples recall the contemporary rituals for diagnosing and chasing away the evil eye or “affascino”. Somehow correlated, a common practice in the classical periods was the employment of olive oil lamps for divination (lychnomancy or lampadomancy), reached through the “interpretation” of the shapes of flames [24].

## 2. Results

### 2.1. From the Latter Half of the 19th Century Until the End of the 1950s

Among the collected sources, forty-one attest to at least one *O. europaea*-based practice used in Italy from the latter half of the nineteenth century until the end of the 1950s, for therapeutic purposes, for averting the evil eye, or for protecting individuals, cultivated fields, dwellings, stalls, and livestock. Collectively, these practices are documented in 90% of Italian regions, with Valle d’Aosta and Trentino Alto Adige representing the only exceptions.

#### 2.1.1. Therapeutic Applications

The study recorded 256 therapeutic preparations incorporating parts of the olive tree, several of which were documented repeatedly in the literature and across different Italian regions. Of these, 184 (72.7%) were complex, multi-component formulations that combined *Olea*-based remedies with botanicals such as ivy (*Hedera helix* L.), wild blackberry (*Rubus ulmifolius* Schott), rue (*Ruta graveolens*), oleander (*Nerium oleander*), onion (*Allium cepa*), savin juniper (*Juniperus sabina*), and St. John’s wort (*Hypericum perforatum*), as well as substances of animal and mineral origin, including ash, sulfur, beaten egg, water, snow, flour, pork fat, and virgin wax. Oral administration (23.4%) and topical application (72.7%) were the most commonly used routes, while 3.5% were unspecified. The plant parts used included oil (87.1%), leaves (5.8%), aerial parts (3.5%), and, in negligible percentages, resin (1.1%), fruit (0.4) and roots (0.4%) (Table 1).

Of the 256 olive tree remedies, 60 (23.4%) were used to treat diseases of the skin and subcutaneous tissue, 42 (16.4%) infectious and parasitic diseases, 37 (14.5%) diseases of the digestive system, 31 (12.1%) injury, poisonings and certain other consequences of external causes, and 18 (7.0%) diseases of the musculoskeletal system and connective tissue (Table 1).

#### 2.1.2. Religious and Magical Practices

The collected data indicate that olive oil, as well as branches and leaves of *O. europaea* L., typically blessed during Palm Sunday, were traditionally employed in Italy between the late 19th and mid-20th centuries to prevent or ward off the evil eye in children [33,58,69,70]. Olive leaves were placed in a small pouch together with incense and grains of salt, which were then sewn into the child’s clothing or positioned among the cradle linens [30,34,44,71]. Another remedy involved fumigating bandages, garments, and even the child or the woman in labor with smoke produced by a brazier containing incense, dried olive leaves, salt, sacred figures, and wax [31,38,66,72]. Finally, to determine whether a fever or headache was caused by the evil eye, the behavior of oil drops poured into a dish was observed [26,27,50].

Within the context of traditional diagnostic practices, the presence of intestinal worms was assessed through a symbolic ritual involving oil and tightly twisted cotton threads. The serpentine movement produced by the twisting of the threads on the oily surface was interpreted as a visible indication of the parasites [35].

Various practices were employed to address different ailments. To remove warts, as many olive branches as there were warts were placed in a stream and anchored to a stone; one was not to pass that place until the branches had fallen [40]. For the treatment of eczema, three sprigs each of nettle (*Urtica dioica*), bramble (*Rubus fruticosus*), and olive were traditionally collected and bundled together. This bundle was then immersed in running water contained within a dish, and the water was subsequently sprinkled onto the affected area [26]. To treat a sty, patients were instructed to gaze into a bottle containing olive oil [49,50]; this same remedy was also believed to prevent headaches and facial blemishes [26,57]. Erysipelas was treated by making three crosses with a silver object and blessed olive leaves [38].

Finally, in the context of ritual remedies documented in Italy between the late nineteenth and early twentieth centuries, olive branches and leaves blessed on Palm Sunday were employed both in agricultural practices and in everyday life. Aerial parts were burned during the summer to quell storms, arranged as crosses at the boundaries of fields to guard against hail, and placed in stables or in rooms used for silkworm breeding to avert malevolent influences [34,46]. Preserved in households, they were displayed at windows during thunderstorms and used to protect people, animals, boats, and objects from fire, the evil eye, witches, and demons [27]. The blessing of bundles composed of olive, palm, and wildflowers extended to crop protection through the strategic placement of sacred fronds in sown fields, vineyards, and olive groves.

Even in maritime contexts, it was believed that the ritual chewing of an olive leaf could ward off the threat of waterspouts [27].

#### 2.1.3. Cosmetic Uses

With regard to cosmetic applications, olive oil treatments were applied to enhance hair strength, shine, softness, and to prevent dandruff [37,44,56].

#### 2.1.4. Syncretism of Contemporary Italian and Ancient Mediterranean Rituals

The celebration of Palm Sunday in Calabria until the early 1900s provided that olive branches adorned with fruit and sweets were brought to the church to be blessed [73], while gifts for the head farmer were hung from an olive branch adorned with bows and flowers during the feast for the end of olive harvest (“*bonifnita*” = well-ended) organized in Trevi (Umbria) [74]. These traditions echo ancient rituals from the classical era. In fact, an olive branch wreathed with wool and laden with fruits and other food-offerings (including olive oil to rub off from the body), called *eiresione*, was carried through the streets of Athens by singing boys during the Pyanopsia festival in honor to Apollo as a symbol of abundance and good harvest (Plutarch, *Theseus* 22, 4–5). A very similar ritual was probably practiced during the Thargelia festival, while a symbolically adorned olive bough called Kobo (so as to hold up an astral system made up of the Sun, the Moon and the stars, following Proclus’s description in *Chrestomathy*, 2nd century CE) was used during the *Daphnephoria*, a Beotian festival—dedicated to Apollo Ismenius or Galaxius—held every ninth year enclosing an initiation rite for young boys from the upper classes.

Moreover, leaves from blessed olive trees in the early 1900s were inserted in small containers, often made of cloth, called *brevi* [75] or *pungas* in Sardinia, and placed around the necks of newborns and children especially. This ritual has a parallel in the *Twelve Books on Medicine* by the 6th century CE physician Alexander of Tralles, describing (XII, 7) the preparation of an amulet against common fever to be hanged around the neck by picking an olive leaf before sunrise and writing with common ink the magic letters κα, ῥοι and α on it. In the same apotropaic regard, the burning of olive branches/logs during the celebrations linked to seasonal cycles and in particular solstices and equinoxes in agricultural-pastoral cultures, has its ancestor in the Roman *Palilia* festival, dedicated to the goddess Pales, protector of shepherds and livestock, when, according to Ovid (*Fasti*, IV 742), it was traditional to burn olive branches for propitiatory purposes. In this vein, the choice of an olive log as *Christmas log* (also called *Yule log* in northern Europe) to be burned on Christmas Eve as a ritual of good luck and prosperity, is attested in Italy in Tuscany where it is called *ciocco* Umbria and Calabria [76]. Eventually, the olive has a certain role even in the protection against the supernatural figures held responsible to trigger atmospheric events. Accordingly, Bellucci [77] in his collection of superstitions related to hail in Umbria, reported that farmers used to shoot rifles at the clouds menacing hailstorms and that the bullets were “reinforced” even with crushed blessed olive leaves and that in some cases the targets were demons, witches or wizards living in the clouds. This tradition is based on the figures of the *Tempestarii*, magicians believed to possess the ability to summon or prevent storms at will, as attested in the 9th century CE treatise titled *Contra insulsam vulgi opinionem de grandine et tonitruis* (*On the absurd popular belief about hail and thunder*) by the bishop Agobard of Lyon. Similarly, Olaus Magnus in the *Historia de Gentibus Septentrionalibus* (1555) wrote that Goths chased away the spirits nestled in the thundering clouds by shooting arrows upwards with their bows. This practice, however, had an illustrious predecessor, considering that according to Cassius Dio, (*Historia Romana*, LIX, 28,6), when it thundered, Caligula threw stones towards the sky.

### 2.2. Third Millennium

Among the collected sources, sixty semi-structured interviews and surveys highlight ethnobotanical knowledge on *O. europaea* documented in Italy at the beginning of the third millennium. This review organizes evidence on medicinal, cosmetic, veterinary, artisanal, domestic, religious/magical, and agro-pastoral uses, focusing on practices involving leaves, oil, fruits, wood, and branches of olive tree. Collectively, these practices are documented in 90% of Italian regions, with Veneto and Trentino Alto Adige representing the only exceptions (Table 2).

#### 2.2.1. Therapeutic Applications

The study recorded 316 therapeutic preparations incorporating parts of olive tree. Of these, 160 (50.6%) were complex, multi-component formulations that combined *Olea*-based remedies with botanicals such as alpenrose (*Rhododendron ferrugineum* L.), old man’s beard (*Clematis vitalba* L.), elderberry (*Sambucus nigra* L.), mountain tobacco (*Arnica montana* L.), garlic (*Allium sativum* L.), borage (*Borago officinalis* L.), and black nightshade (*Solanum nigrum L.*), as well as substances of animal and mineral origin, including ash, water, wine, salt, flour, pork fat, and beeswax. Oral administration (26.9%) and topical application (69.9%) were the most commonly used routes, while 3.2% were unspecified. The plant parts used included oil (77.4%), leaves (19.6%), aerial parts (1.5%), and, in negligible percentages, resin (0.3%), fruit (0.3%), bark (0.3%), seed and oil (0.3%), and fruit and oil (0.3%) (Table 2). Of the 316 olive tree remedies, 86 (27.2%) were used to treat diseases of the skin and subcutaneous tissue, 46 (14.6%) diseases of the digestive system, 41 (13.0%) diseases of the circulatory system and connective tissue, 29 (9.2%) diseases of the ear and mastoid process, and 26 (8.2%) injury, poisonings and certain other consequences of external causes (Table 2).

#### 2.2.2. Religious and Magical Practices

The collected data indicate that olive oil is still employed in Italy to prevent or ward off the evil eye [80,83,99,104]. Olive oil is also used in religious practices to trace the sign of the cross on the forehead to alleviate pain [80,114], while oil obtained from the votive lamp dedicated to Saint Biagio is employed by fishermen to treat sore throats [104]. Leaves and branches are blessed in church on Palm Sunday [95,115]. These leaves and branches, sometimes arranged in the form of a cross, are used to protect dwellings and agricultural fields from harmful entities or as symbols of good fortune [81,132], as well as to safeguard crops from adverse weather events such as hailstorms [99]. Finally, branches of *O. europaea* are held in the hand while praying to Saint Antonio to help locate lost objects [99].

#### 2.2.3. Cosmetic Uses

Within the context of cosmetic uses, olive oil is employed to strengthen hair and enhance its shine [80,81,95,99]. It is also used to moisturize the hands, either applied alone, in combination with lemon juice [95], or mixed with shoots of *Sambucus nigra* [99]. Additionally, olive oil is applied for body care [90] or blended with fruit juice for tanning purposes [96].

#### 2.2.4. Domestic Uses

*O. europaea* is documented as having a wide range of traditional domestic uses, reflecting the versatility of its different plant parts. The wood and branches are reported to be used as firewood for lighting fires and heating ovens, although they are not generally employed for cooking [99,104]; in some cases, their use is associated with the production of bread with a characteristic aroma [99]. Residues derived from olive oil extraction, such as olive husks, are likewise utilized as domestic fuel [84]. Olive wood is frequently cited as suitable for the manufacturing of furniture, household utensils, carpentry tools, and various traditional objects, including toys and implements connected with rural domestic activities, such as tools employed during pig slaughtering [98,104,115,133]. Branches, suckers, young shoots, and stems, valued for their flexibility, are commonly used in basketry and handicraft production, either alone or in combination with other plant species (e.g., *Arundo donax*, *Clematis vitalba*, *Castanea sativa*) to produce containers and braided objects for domestic use [96,104,114,121,134,135]. Olive oil is consistently reported as a key domestic resource; it is employed as lamp fuel and in the preparation of detergents and soaps, often derived from low-quality oil or by-products of olive mills [90,95,99,104,114]. Furthermore, olive oil is used in household practices related to food management, including techniques aimed at advancing fruit ripening, such as pricking figs with a needle dipped in oil [89,118]. Additional domestic uses reported in the literature include the preparation of traditional liquors from olive leaves [122] and ethnographically recorded disciplinary practices involving young shoots of wild olive (*O. europaea* var. *sylvestris*) [115].

#### 2.2.5. Veterinary Uses

Uses of *O. europaea* are associated with a diverse range of traditional practices aimed at managing animal health, primarily through the use of olive oil and, to a lesser extent, its processing residues. Olive oil sediment (“morca”) is described as having been applied topically, often in combination with pork fat or sulfur, to wounds in sheep and cattle with the purpose of repelling flies [86]. Olive oil itself is further reported to have been used in simple topical applications for the removal of ticks [86] and, when combined with sulfur, for the local treatment of scabies in dogs, cats, and rabbits [103]. In relation to digestive and abdominal disorders, olive oil appears to have been administered orally, mixed with salt and vinegar in cases of bovine tympanism [104], or combined with milk as a purgative for cows and goats [95]. Several external applications are also documented: olive oil served as a carrier or base for preparations involving other plant species, such as dried and minced galls of *Rhododendron ferrugineum* used as plasters for minor fractures [124], or *Senecio vulgaris* cooked in oil and beeswax to obtain ointments described as anti-inflammatory [80]. Macerations of *Hypericum perforatum* aerial parts in olive oil are reported to have been massaged onto swollen teats of goats and onto the abdomen of parturient animals, with the aim of reducing inflammation and facilitating placental expulsion [90]. Similarly, leaves of *Ruta graveolens* fried in olive oil were applied externally to swollen abdomens of animals after parturition [90]. Additional uses reported in the literature include the topical application of olive oil to animals experiencing hair loss [99], its use as a carrier for preparations of *Laurus nobilis* applied to the animal coat as a fly repellent [134], and its function as a solvent for macerations of *Allium sativum* bulbs described as being employed in the treatment of poultry diseases [93]. Furthermore, olive oil is documented as being used in the management of digestive conditions in cattle, where it accompanies decoctions of fresh leaves of *Rumex alpinus* [124].

#### 2.2.6. Zootechnical Uses

Leaves and young stems are used as winter fodder for sheep and goats [84,104].

#### 2.2.7. Syncretism of Modern-Day Italian and Ancient Mediterranean Rituals

Celebrations in which the symbolism of *eiresione* continues to live in the present day, absorbed in a different religious context, include the feast of Palm Sunday in the Sorrento Peninsula where blessed olive branches are decorated with caciocavallo cheese, sweets and ribbons and carried in procession, and in the offering to Saint Rocco of olive branches decorated with typical local artisanal sweets in Lettomanoppello (Abruzzo) on the first Sunday of October. A further intersection between Christianity and paganism finds expression in the Palm Sunday celebrations of Bova, a village of the “Grecanic” area of Calabria, in which the ancient Eleusinian mysteries dedicated to Demeter and Persephone, linked to the cyclic change in seasons and agriculture, are entwined with the Catholic rites. During this feast, female puppets, the “Pupazze” or “Persephoni”, made of olive leaves woven around a pole and decorated with flowers, ribbons and sweets are carried in procession along the streets of the town. At the end of the procession, the cut olive “twigs” are blessed in church and are distributed to the faithful to apotropaically replace those from the previous year which can be burned to drive away evil. A Persephone-Kyrà Sarakostì (the Byzantine figure of the “Lady Lent”) syncretism has also been proposed [136].

## 3. Discussion

Ethnobotanical knowledge, encompassing the understanding of plant properties and uses represents a fundamental component of cultural heritage of indigenous and local communities and underpins ethnobotany, the discipline that examines the dynamic relationships between human societies and plant resources across time [137].

Over millennia, coastal regions of the Mediterranean and the Near East have evolved into intricate socio-ecological systems shaped by long-standing practices such as agroforestry, pastoralism, and diversified small-scale farming. Within this context, the olive tree, together with wheat and the vine, may be regarded as emblematic of a shared agrarian Mediterranean civilization, reflecting a collective human capacity to shape the physical environment in ways profoundly influenced by both climatic conditions and historical processes [138] thus leaving enduring marks on the cultural dimension of Mediterranean societies.

From a cultural perspective, *O. europaea* occupies a central place in Italian ethnobotanical heritage, contributing to healthcare practices, nutrition, ritual activities, and numerous everyday applications. Recent field-based studies have highlighted a notable persistence of ethnobotanical knowledge, particularly within a context characterized by the clear coexistence of traditional practices and more “modern” uses [139].

This work highlights the continuity of *O. europaea*-based remedies in the treatment of various ailments, which were employed in Italian folk medicine from the late 19th to the early and mid-20th centuries, as well as at the beginning of the third millennium. Considering the millenary roots of the ethnobotany of *O. europaea*, the biomedical categorization of folk remedies reveals remarkable continuity in how human illnesses were targeted. Diseases of the skin and subcutaneous tissue remained the primary therapeutic target, utilizing the emollient, barrier-repairing properties of olive oil. Gastrointestinal and external injuries also remained top-tier indications across centuries. A distinct modern addition is the emergence of remedies targeting diseases of the circulatory system, demonstrating how folk medicine adapts to and mirrors the epidemiological shifts (such as cardiovascular conditions) of modern populations. 

The effectiveness of these treatments could be related to diverse pharmacological activities, exhibited both in vitro and in vivo, such as antidiabetic, antioxidant, anticancer, and wound-healing properties [140]. In the same way many treatments are still used for veterinary purposes.

From an anthropological perspective, blessed leaves, branches, and oil are included among the Sacramentals, which are instituted for the sanctification of specific ecclesiastical ministries, particular states of life, diverse situations within Christian experience, and the use of various elements beneficial to human well-being [141]. Remedies employed in Italian folk traditions often regarded consecrated leaves, branches, and oil as powerful symbols of rebirth, capable of eliminating sin and warding off evil, which was frequently considered the cause of illness and misfortune [142].

Furthermore, the attribution of specific healing capacities to saints—such as rubbing the throat with the oil of Saint Biagio—appears to be closely linked to events traditionally associated with their lives. In this context, Saint Biagio came to be regarded as an intercessor for throat disorders, a role commonly understood to derive from a hagiographical tradition according to which he saved a child at risk of choking [142]. Similarly, the long-established custom of invoking Saint Antonio of Padua for the recovery of lost objects is generally traced to an episode said to have occurred in Montpellier, where he reportedly recovered a psalter taken by a novice through the efficacy of prayer. Over time, such narratives appear to have contributed significantly to shaping devotional practices associated with these saints. At the same time, magical remedies were also employed, such as massaging the painful area with olive oil in which a hot iron had been immersed, in an attempt to transfer the healing power of iron, believed capable of driving away the evil spirits afflicting the patient [143].

This should not be regarded as surprising. At present, it seems that a portion of the Italian population continues to turn to traditional healing practices, using amulets, and performing rituals accompanied by the recitation of specific formulas and orations. These practices are often associated with the treatment of conditions such as the *malocchio* (evil eye), which notably ranked among the most frequently searched terms on Google Italy in 2014 [142].

Generating a rich, inclusive dataset to develop a comprehensive olive-based monograph can be indispensable to understand (cultural patterns of) plant knowledge, as opposed to a reductionist (and inevitably incomplete) approach. The re-evaluation and utilization of this knowledge might be of potential value in connection with the safeguarding of intangible cultural heritage as promoted by UNESCO (www.unesco.org; accessed on 2 June 2026).

Moreover, it may have important implications in terms of the protection of the rights of holders of traditional knowledge, as defined in the Convention on Biological Diversity (www.cbd.int; accessed on 2 June 2026) and embraced by the Intergovernmental Committee on Intellectual Property and Genetic Resources, Traditional Knowledge and Folklore of the World Intellectual Property Organization (http://www.wipo.int/tk/en/igc/; accessed on 2 June 2026).

Across centuries, the coastal landscapes of the Mediterranean and Near East have developed as intricate socio-ecological systems that have not only sustained biodiversity but have also enabled the intergenerational transmission of place-based knowledge [144].

Despite the spread of monocultural farming systems in areas most suited to intensification and, conversely, the abandonment of cultivation in marginal zones, olive-growing systems and landscapes continue to represent one of the defining features of the Italian historical landscape [145].

At present, approximately 35% (12 out of 34) of the Italian historical rural landscapes listed in the National Register of Rural Landscapes of Historical Interest are represented by olive groves and traditional olive-growing systems, which are primarily distributed across the central and southern regions of the country (Tuscany, Liguria, Umbria, Latium, Campania, Apulia, Calabria, and Sicily) [146]. Moreover, given their environmental and cultural relevance, the conservation of centuries-old olive trees and olive orchards of particular agronomic and landscape value is established in the National law 14/01/2013 no. 10 (article 7) and in the Regional laws (R.L.) of Calabria (R.L. 30/10/2012 n. 48 “Protection and enhancement of the olive-growing heritage of the Calabria Region”), Basilicata (R.L. 06/08/2015, n. 24 “Rules for the protection, enhancement and promotion of regional olive growing and regulations for the felling and cutting of olive trees”) and Apulia (R.L. 06/04/2007 n. 14 “Protection and enhancement of the landscape of monumental olive trees in Apulia”). Not surprisingly, 75 olive or olive oil-based products have been classified as TAPs (Traditional Agrifood Products), in accordance with the provisions contained in the Legislative Decree 173/1998, aimed at protecting the historical memory of an agricultural product or a traditional dish [147].

Nevertheless, the progressive erosion of ethnobotanical heritage has emerged as a major concern in the region, particularly in relation to the so-called “Hysteresis Effect,” whereby lost knowledge systems become difficult, if not impossible, to restore even when favorable conditions for their recovery reappear [144].

In recent decades, the Mediterranean region has undergone profound socio-environmental transformations. Since the mid-twentieth century, processes such as rural depopulation, agricultural intensification, climate-related pressures, and the rapid expansion of mass tourism have significantly disrupted long-standing relationships between human communities and their environments [148,149]. As a consequence, local knowledge systems have experienced accelerated decline. The socio-ecological consequences of this process are increasingly visible in the form of deteriorating terraces, neglected groves, and fragmented knowledge traditions preserved only in the memories of older generations, symbolic cultural practices, or commodified representations aimed at tourists. In this regard, studies assessing the effects of climate change on olive orchards indicate a geographic northward shift in Italian olive cultivation, with decreases at varying rates in Central and Southern Italy due to widespread land abandonment [150,151]. This decline is driven by diminishing profitability in marginal areas [152], due to lower productions, with a negative trend that could consolidate in the next few years because of reduction in winter chilling units and significant earlier phenological development [153]. This reduction in rural olive-growing areas inevitably takes away a part of the traditional popular knowledge and cultural heritage of Southern Italy especially. The risk of genetic (and cultural heritage) erosion has been further exacerbated in the Apulia region (the first Italian olive producer Region) by the outbreak of *Xylella fastidiosa*, the pathogenic bacterium responsible for the ‘olive quick decline syndrome’ (OQDS), that caused the felling of approximately 17,000 trees during the 2013–2024 period [154] most of which were centuries-old, monumental olive trees.

## 4. Materials and Methods

Relevant literature about the use of *O. europaea* L. in Italian traditional ethnobotanical knowledge was gathered by searching the National Library Service website of the Italian Libraries Network using keywords such as “medicina popolare” (traditional medicine), “rimedi popolari” (popular remedies), “usi e costumi (usages and customs), “tradizioni popolari” (traditions), “cultura tradizionale” (traditional knowledge). This approach yielded approximately one hundred primary sources, including books and journal articles authored by anthropologists, physicians, ethnographers, folklorists, and local history scholars, dating from the latter half of the 19th century to the first half of the 20th century. This era marks the inception of a more systematic and academically rigorous approach to investigating folk traditions, particularly those involving remedies for various diseases [155]. The 1950s, in contrast, marked a significant turning point in Italy’s economic prosperity, characterized by extensive rural-to-urban migration and a shift toward industrialization. For the first time in Italy’s history, the number of individuals employed in industrial sectors surpassed those working in agriculture [156], marking a significant transformation from a predominantly rural society to a modern, industrialized nation [157].

**Figure 1:**
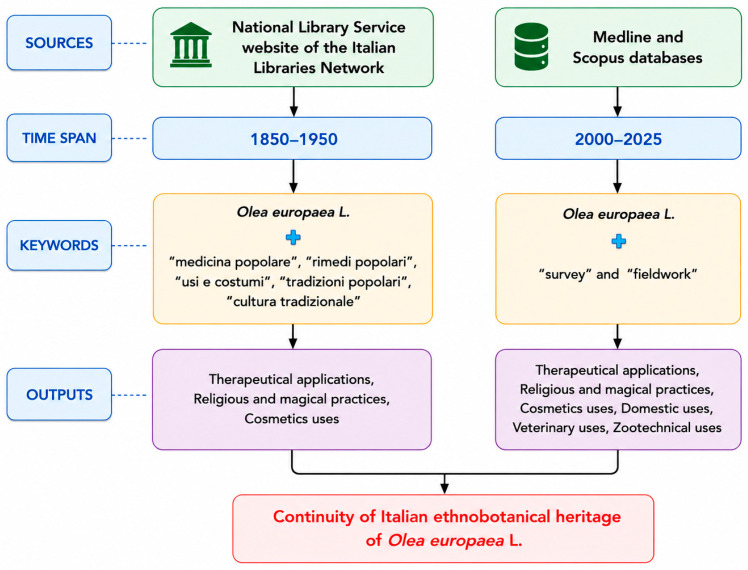
Flowchart of the literature search and data selection comparing historical (1850–1950) and contemporary (2000–2025) sources on the ethnobotanical uses of *Olea europaea* L. in Italy.

Subsequently, a literature search was conducted in Medline and Scopus to identify Italian ethnobotanical studies published between 2000 and 2025. The search combined *O. europaea* L. with the keywords “survey” and “fieldwork” to assess whether this traditional ethnobotanical knowledge is still in use. For each of the aforementioned periods, the collected data were categorized into “therapeutic applications”, “religious and magical practices”, and “cosmetic uses” (Figure 1). Diseases and disorders included under *therapeutic applications* were classified according to the World Health Organization’s *International Classification of Diseases* [158]. Furthermore, for the period 2000–2025, the following additional categories of remedies were included: “domestic uses”, “veterinary uses”, and “zootechnical uses”.

## 5. Conclusions

*O. europaea* L. represents a fundamental cornerstone of Italian biocultural heritage. Its traditional use is almost universally distributed across the Italian peninsula, consistently spanning 90% of the country’s regions across both historical and contemporary eras. The enduring presence of these practices underscores the plant’s profound integration into rural Italian life, transcending simple agricultural utility to influence medical, domestic, and spiritual spheres.

However, contemporary practices reflect a notable modernization of folk medicine, likely influenced by contemporary scientific validation of the antioxidant and cardiovascular benefits of the chemical composition of olive oil and leaves. The data highlights a highly sophisticated intersection between pragmatic rational medicine and magico-religious ritualism: the same plant parts could be used to treat dermatological conditions or, via fumigation, amulets, and symbolic gestures, to ward off metaphysical threats like the “evil eye” (malocchio).

Nevertheless, the progressive erosion of ethnobotanical heritage, including both the intangible corpus of traditional knowledge and the agricultural systems through which such knowledge is maintained and transmitted, has emerged as a major concern in the face of contemporary challenges such as climate change and phytosanitary crises. Preserving and documenting this knowledge is therefore essential not only for safeguarding cultural identity but also for maintaining a valuable reservoir of biocultural diversity and potentially useful ethnopharmacological information.

## Figures and Tables

**Table 1:** Traditional uses of *Olea europaea* L. to treat diseases in Italy documented from the latter half of the 19th century to the end of the 1950s, based on historical and ethnobotanical literature.

Remedy	Pathology	Administration	Reference
**Certain infectious and parasitic diseases**			
Olive oil	Worms	Children with fair skin and blond hair are said to have “worm-like complexion,” and it is believed that ascarids more easily lie in wait for them than for those with darker skin; for this reason such children are subjected to continual prophylactic measures. From time to time they are given, for internal use, either the inner membrane of an egg, dried on a red-hot iron shovel and ground into powder, or scorched eggshells mixed with sugar and olive-shoot juice; and externally, their navel is rubbed with pig excrement dissolved in hot oil, or they are made to constantly wear around their neck a necklace made of the vertebrae of a snake that has died and been eaten clean by ants.	[25]
Olive oil	Pleurisy/pneumonia; a wonderful remedy for the sting (ponture)	Sweet common oil, 4 ounces, boiled with 8 ounces of common water—but it is better to use blessed thistle water if available—until the water has evaporated. It is drunk warm. I have tried it several times with successful results.	[26]
Olive tree	Gonorrhea	A decoction of ivy (lènece), bramble shoots (tanne de ruve), buds (pòppe) of the gentle olive tree—one that has not yet borne fruit—licorice roots (recùlizìe), pellitory (jèrva murène), and mallow are beneficial. Boil these herbs in four jugs of water until more than half have evaporated; one glass is taken in the morning.	[26]
Olive oil	*Cholera* (*culera*)	Olive oil with lemon, with the intention of refreshing the stomach.	[27]
Olive tree	Malarial fever	Decoction of leaves.	[28]
Olive tree	Tertian fever	Take the tips of wormwood (*Artemisia absinthium*), olive shoots, horehound (*Marrubium vulgare*), garlic, and willow bark (*Salix*); make a decoction of them, then mix it together with strong wine to be taken two hours before the fever attack.	[25]
Olive tree	Tertian fever (malaria)	Wild olive, known as the oleaster.	[29]
Olive tree	Malaria	Decoction of olive leaves, sage, and tomato.	[30]
Olive tree	Malaria	Leaf infusions.	[31]
Olive tree	Intermittent fevers	In the district of Palmi, before 1842, our people used the powder and decoction of yarrow flowers to treat intermittent, periodic, and autumnal fevers; as a remedy it was considered more effective than preparations of quinine, along with olive leaves, garlic.	[32]
Olive oil	Dog bite	Ointments are made using dog hair fried in oil.	[33]
Olive oil	Hydrophobia (*raggia*)	Some people apply dog hair directly to the wound, after soaking it in oil; and if the oil is old, it is considered in itself to possess anti-rabic properties.	[27]
Olive oil	Mumps (incordatura del collo)	The treatment consists of anointing or rubbing the affected area with hot oil, then applying fomentations and bandaging the neck with cotton wool.	[34]
Olive oil	Tetanus	While waiting for the doctor, the wound is anointed with warm oil.	[34]
Olive oil + ritual	Helminthiasis	In order to diagnose the presence of worms, the midwife (commare) pours some oil into a plate and tightly twists together cotton thread, four- or eight-ply, then throws that sort of wick onto the oil. Naturally, the strongly twisted threads unwind with greater or lesser speed, and the serpentine movement of the fibers on the oily surface produces an effect on the onlookers, who believe they see in it the worms driven out from the child’s gastro-enteric tract.	[35]
Olive oil	Helminthiasis	To eat lemon with oil and salt.	[36]
Olive oil	Helminthiasis	Squeezed lemon, a spoonful of olive oil, and wheat flour, mixed to form a soft paste to be given for three mornings on an empty stomach.	[37]
Olive oil	Helminthiasis	A cooked mash made with garlic and oil.	[37]
Olive oil	Helminthiasis	Lemon, especially when mixed with olive oil.	[27]
Olive oil	Helminthiasis	An anointing made with half a glass of olive oil boiled with three cloves of garlic, applied to the trachea, the stomach, and the soles of the feet.	[33]
Olive oil	Helminthiasis (*virminaca*, *vermi*, *maschi o maschuni*, *mancuni*, *scuminicati*)	They drink olive oil with lemon, or even pure olive oil.	[27]
Lamp oil	Erysipelas	Its most frequent use is in rheumatic pains, and in pains of any other kind; at the onset of erysipelatous and phlegmonous inflammations of the skin, as well as in adenitis.	[26]
Olive tree/olive oil + prayer	Erysipelas	With a black hen’s feather dipped in oil, the affected part is touched while saying a general prayer, although this almost always varied. Along with the black hen’s feather, an olive leaf is used, and both are dipped in oil, with which the erysipelas is anointed, and then another oration is said. It is understood that these prayers must first be recited in church three times each year, always on Christmas night; indeed, they are not considered effective unless they are then recited for three consecutive days, twice a day, that is, before sunrise and after sunset.	[33]
Olive oil + prayer	Erysipelas	With a tuft of wool dipped in oil, the affected part is touched and an oration is recited. It is understood that these prayers must first be recited in church three times each year, always on Christmas night; indeed, they are not considered effective unless they are then recited for three consecutive days, twice a day, that is, before sunrise and after sunset.	[33]
Olive tree	Erysipelas	One takes a leaf of olive blessed on Palm Sunday, and a piece of silver, and says an oration. At the same time, three crosses are made over the afflicted area. This charm, too, must be performed at sunset.	[38]
Olive tree/olive oil + spell	Erysipelas	A spell is recited. After this, with a black hen’s feather dipped in oil, the inflamed part is anointed first around the edges, then in the form of a cross. When the anointing has been done with an olive twig, the area must then be moistened with the blood of a black hen.	[26]
Olive oil + secret word	Erysipelas	A poultice of lettuce and warm oil is applied, and then a gold or silver coin is passed all around the edge of the patch, while secret words are pronounced at the same time.	[39]
Olive oil	Whitlow	Another “maturing” poultice is made with fine flour, water, oil, wine, and salt.	[26]
Olive oil	Verminous ulcers	These sores are treated by anointing them with warm oil and soot.	[36]
Olive tree	Cutaneous warts (porru)	Warts disappear from the hands or other areas by placing them beneath a stone in a running stream with as many olive branches as there are warts. Until they have fallen off, one must not pass by that place (Vena).	[40]
Olive oil	Scabies	It is still used in our countryside to remove scabies using an ointment made with Sabine leaves and olive oil.	[25]
Olive oil	Scabies	Rubbed with oleander fronds, fried in oil and finally washed with lye.	[33]
Olive oil	Scabies	Anointments with ointment composed of five sous of sabatina, a sou of niter, one of salt and half a pound of oil; afterwards usual washing occurs.	[33]
Olive oil	Scabies	Anointing with an ointment composed of sulfur, saltpeter and oil and finally washed.	[33]
Olive oil	Scabies	Ointment composed as follows: juice of ten lemons and crushed and sieved funicillo root peel are boiled with sweet olive oil and pork fat; a handful of white hellebore and a handful of salt are added; this is then rubbed for several evenings and finally washed.	[33]
Olive oil	Scabies	Ointment of oil, sulfur and white fig ash rubbed with chickpea plants.	[33]
Olive oil	Scabies (*rugna*)	Local rubs of lemon juice, oil and sulfur.	[27]
Olive oil	Scabies	Scabies is generally divided into two categories: in the first the process appears in rather large patches; in the second in small patches. For this disease, folk therapy provides us with a complete recipe made up of horseradish roots, six fifths of olive oil, half a fifth of lard, three lemons, a penny of vinegar and a pinch of ash. The rind is removed from the roots, crushed well in a mortar, placed in a new pan and boiled three or four times. This recipe, sufficient for four people, is given for the ordinary scabies of the first category. For the second category, known as dog scabies, a little canine onion (Seyila marittima) is added to the above. In the evening, before going to bed, the preparation is reheated and ample rubs are made over the body, taking care not to grease certain parts. Three days of treatment, done alternately, are sufficient for healing.	[41]
Olive oil	Dermatophytosis	To anoint the scabs with oil in which lemon verbena has been infused. Today, many replace ordinary oil with cod-liver oil.	[34]
Olive oil	Dermatophytosis	To anoint the scabs with oil in which St. John’s wort has been infused. Today, many replace ordinary oil with cod-liver oil.	[34]
Olive oil	Herpes labialis (*focu alestru*, *focu arestu*, *sfogu di frevi*)	It is healed with oil and wax melted together.	[27]
Olive tree	Antimalarial		[42]
**Neoplasm**			
Olive oil	Breasts (tumor)	As soon as suppuration has occurred, a poultice of oil and virgin wax is applied.	[33]
**Endocrine, nutritional and metabolic diseases**			
Olive oil	Spleen ailment	Anointing the area with warm olive oil in the evening, then the following morning washing it with one’s own urine, which must also be drunk.	[33]
Olive tree	Splenic hepatitis (*u mali di l’arcu*)	A decoction of olive leaves.	[43]
Iron-infused oil	Gout	Applications of ‘iron-infused oil’ to the joints and to the affected areas.	[44]
**Mental and behavioral disorders**			
Olive tree + incantation	Hysteria	Uttering an incantation (a charm) with a witch-like ritual, using a few grains of incense, a pinch of salt, and some blessed olive leaves burned over glowing coals, producing a smoke which, accompanied by the muttering of mysterious words, is believed to be capable of working the miracle.	[35]
Olive tree	Nervousness	Fumigations made from leaves, with the addition of feathers from any animal.	[45]
**Diseases of the nervous system**			
Blessed olive tree	Headache	Two blessed olive twigs, placed crossed behind the head, make the headache go away.	[46]
Olive oil	Headache	If the headache comes from overheating or from rheumatism, the head is anointed with common oil; then some well-beaten egg white is taken and placed on a bit of virgin tow, which is then tied under the soles of the feet when going to bed: this draws the blood and the heat downward, and one feels better at once. Some, instead of egg white, place crushed snails under the soles of the feet.	[47]
Olive oil	Headache	Slices of lemon are applied to the temples, first dusted with roasted coffee powder, which acts as a kind of vesicant, and the head is also anointed with common oil.	[47]
Olive oil	Headache	Another preventive measure is to look at oneself in the same morning in a ‘mirror’ of oil, just as one does when the first hairs begin to appear.	[26]
Olive oil + prayer	Sunstroke (*chiovu sulariu*, *chiovu di suli*, *suli ‘n testa*, *corpu du suli*, *botta di suli*)	To calm the headache—or, as they say, to remove the sun from the head—a plate is taken, a little oil is poured into it, and, placing it on the head, a prayer is recited while making the sign of the cross. As for the drops of oil, it is noted that there must be three of them, which serve for the sure diagnosis of the ailment. If they spread out over the plate, it is a case of sunstroke; if they do not, it is not.	[27]
Olive oil	Sciatica	A miraculous cure of sciatica: Pè d’oca (fresh leaves), Pè d’ nader, Lèngua ed can, Romsa (these ingredients in a quantity that fits in one hand), Fava flour (100 g), Mustard flour (100 g), half a small bottle of Sloan’s Liniment at 10 degrees, a glass of wine with an alcohol content above 10 degrees and 4 tablespoons of vinegar. All of this must be boiled in a bain-marie over a low flame for fifteen minutes. Then let it cool, and add 4 egg whites and the remaining half of the Sloan’s Liniment used previously; mix well and spread the poultice onto a cloth sprinkled with mustard flour, and apply the resulting plaster to the painful area, leaving it on for exactly one hour. Once the application is completed, the area must be anointed three or four times over the following twenty-four hours with another ointment prepared by boiling and then straining through cloth: one glass of olive oil, 100 earthworms from moist soil, one quarter of a glass of horse chestnuts cut into very thin slices.	[48]
Iron-infused oil	Sciatica	Iron-infused oil.	[44]
Olive oil	Sciatica (*siatica*)	The usual anointings and rubbings with warm oil, petroleum, etc.	[27]
Olive tree + incantation	Seizures	Uttering an incantation (a charm) with a witch-like ritual, using a few grains of incense, a pinch of salt, and some blessed olive leaves burned on glowing coals, producing a smoke which, accompanied by the murmuring of mysterious words, is believed to be capable of working the miracle.	[35]
Olive oil	Neuralgia (*dulura nirvusi*)	Ointment with olive oil.	[27]
**Diseases of the eye and adnexa**			
Olive tree	Eye pain	Some people use two egg whites, placing a little shaving of an olive branch inside them and laying them over the eyes, which should then be covered before going to bed.	[47]
Blessed olive oil/blessed lamp oil	Eye inflammation	The remedy considered the best is to anoint the eyes with blessed oil, especially with the oil that burns in the lamps before an altar of Saint Francis.	[25]
Olive oil	Stye	To look at the mouth of the oil flask and carefully observe its contents.	[49]
Olive oil	Stye (‘u rijiuòlo)	First of all, it must be ‘calmed’ three times by making the sign of the cross over it with a ‘male’ key or with the eye of a needle; and then the sick person, until complete recovery, must look every morning for a good while into a bottle containing olive oil.	[50]
**Disease of the ear and mastoid process**			
Olive oil	Ear	To put warm glycerin, or warm oil or milk, into the ear, then plug the ear and bandage it, placing beneath the bandage a layer of, preferably oiled, paper.	[44]
Olive oil	Otitis/otalgia	Small underground mushrooms (pan porcini), which appear as little balls about the size of two lire coins with leaves similar to clover, are gathered. They are filled with olive oil, and boiled as a whole. This oil, very warm, is then applied inside the ear using cotton soaked with it.	[49]
Lamp oil	Abscess (postiema)	One goes to a woman who is nursing a male child, and has a few drops of her breast milk squirted into the ear; or she gives some milk which, mixed with a little oil from a consumed lamp, is placed inside the ear with a bit of cotton, which is changed from time to time.	[47]
**Diseases of the circulatory system**			
Lamp oil	Hemorrhoids	The area is anointed with lamp oil, taking care to use the thick green residue that settles at the bottom.	[49]
Olive oil	Hemorrhoids	Cloths soaked in wine and sweet olive oil are applied.	[33]
Iron-infused olive oil	Hemorrhoids	Anointings with ‘iron-infused oil’.	[44]
Olive oil	Hemorrhoids (*murròiti*, *testi di vini*, *capi-vini*, *vini*, *vini abbàsciu o nisciuti*	Fumigations of oil poured onto glowing coals at the bottom of a vessel.	[27]
**Diseases of the respiratory system**			
Lamp oil	Nasal pain	To rub it with warm lamp oil.	[37]
Iron-infused oil	Aphonia, dysphonia, hoarseness	Use of applications of iron-infused oil on the front of the neck, followed by the application of tow, or of warm ashes wrapped in a handkerchief.	[26]
Blessed olive oil	Catarrhal/diphtheric angina	Against catarrhal or diphtheric angina, oil that is blessed on St. Blaise’s Day is used.	[30]
Lamp oil (*òjje de la lume*)/iron-infused oil	Tonsillar angina	Gargles made with a decoction of barley and vinegar are helpful. Also useful are applications of ‘lamp oil’ or iron-infused oil, followed by the application of tow, or small bags of warm ash.	[26]
Olive oil	Sore throat/pharyngitis	The throat is rubbed with olive oil, butter, or chicken fat, and warm ash wrapped in blotting paper or in a piece of red wool is applied over it.	[33]
Olive oil	Sore throat/tonsillitis	Applications of oil beaten together with chamomile water.	[33]
Lamp oil	Nasal pain	Whether the pain is on the outside or inside, it is rubbed with lamp oil, and it heals immediately.	[47]
Olive oil + oration	pneumonia/pleuritis	Leeches are applied on the back, then the area is rubbed with oil and the right thumb is pressed and slid firmly over it while saying an oration.	[33]
Olive oil	Cold (asthma)	A footbath with wine is given, then the feet are rubbed with oil or honey mixed with salt.	[33]
Olive oil	Cold (children)	When breathing becomes difficult, warm-oil rubs are applied to the nostrils.	[41]
Lamp oil	Braying cough	One would take the child to the Madonna of the Cough near the Stagno bridge (Lastra a Signa) and take some of the oil from the small lamp to anoint the child’s throat, and put a few drops into the child’s food once back home. Naturally, one must pour fresh oil into the lamp and make an offering to the Madonna. On the way back, it is advisable to take a road that is particularly difficult to travel.	[51]
**Diseases of the digestive system**			
Olive oil	Acidity (*acitu*)	Drink boiled water with olive oil.	[27]
Olive oil	Colic	Half a small glass of wine, half a small glass of fine Lucca olive oil, some good pepper, and two slices of bread are cooked together. You eat it, and the pains disappear.	[47]
Olive oil	Colic	Half a glass of oil, a tablespoon of pepper, and the juice of two lemons; mix it and drink it.	[47]
Olive oil	Abdominal colic	Rubbings of warm oil on the abdomen or enemas.	[28]
Olive oil	Intestinal colic (*colica*, *duluri colicu*, *viddicu cadutu*)	An anointing with oil performed using the little finger of the right hand.	[27]
Olive oil	Intestinal colic (*colica*, *duluri colicu*, *viddicu cadutu*)	Hot water with olive oil mixed in is given to drink.	[27]
Olive oil	Abdominal colic	The sick person is made to drink some oil, and then three crosses are traced, one on the sternum, one on the navel, and one above the pubis.	[34]
Olive tree	Dental caries (*denti* o *ganga fradicia*, *denti puritu*, *ganga purruda*)	A few small pieces of olive gum are inserted into the cavity of the carious tooth.	[27]
Lamp oil	Abdominal pain	To relieve the pain and to facilitate the expulsion of gas and intestinal contents, a very common remedy is to take three drops of ‘lamp oil’ in a teaspoon of milk.	[26]
Olive oil	Abdominal pain	After crushing a clove of garlic, two measures of olive oil, one-fifth of water, and a spoonful of salt are added. The mixture is given to drink cold.	[52]
Olive oil	Hernia	Eating foods rich in oil is believed to promote the development of hernias; and therefore, since religious men are for the most part affected by this ailment, it has been thought that this occurs as a consequence of their constant abstinent diet. Thus, a person with a hernia is disallowed by women to take oily purgatives, because these, according to them, might cause the hernia to protrude even further.	[25]
Olive oil	Indigestion	To induce vomiting, one had to drink some lukewarm water mixed with a few drops of oil.	[53]
Olive oil	Indigestion	Ordinary olive oil.	[34]
Olive oil	Enteritis (*infiammazione ai budelli*)	As a purgative, mallow water mixed with olive oil was taken.	[25]
Olive oil	Infantil colic (*latti aggruppatu* o *addugghiatu*, *latti crucïatu)*	It is common practice to apply oil rubs on the baby’s abdomen.	[27]
Olive tree	Toothache	A piece of olive gum is used to carefully seal the cavity hole.	[25]
Olive tree/olive pit	Toothache/dental caries	Olive gum or olive pit kernel.	[33]
Lamp oil	Toothache/dental caries	A small wad of hemp or linen cloth soaked in oil and warmed over the flame of an oil lamp.	[33]
Olive oil	Abdominal pain	A couple of finger-widths of olive oil in half a glass of mallow water is drunk.	[37]
Olive oil	Abdominal pain	A tablespoon of oil with vinegar and a pinch of salt.	[54]
Olive oil	Abdominal pain in children	The abdomen was anointed with oil.	[34]
Olive oil	Abdominal pain in children	A poultice made of oil and tobacco.	[34]
Olive oil	Abdominal pain in children	They put regular oil in the baby food, because it was considered effective against abdominal pain.	[47]
Olive oil	Abdominal/stomachache	A beverage of crushed onion juice, tempered with a bit of oil.	[33]
Olive oil	Stomachache	Oil rubs.	[34]
Olive oil	Stomachache	An old doctor advises treating stomach colic by taking 40 g of olive oil mixed with a small glass of wine vinegar at the moment the attack begins.	[32]
Olive oil	Abdominal pain	To cure abdominal pain, equal parts of ‘erba de vientu’, warm oil, and vinegar are mixed together and drunk.	[55]
Olive oil	Prolapse of the rectum	A mixture of oil and ash from old cloths was made, and the rectum was anointed with it.	[34]
Olive oil	Purgative	As a purgative, people used two finger-depths of olive oil in a glass; to make it less nauseating, they added the juice of half a small lemon. Sailors, instead of lemon, used seawater.	[56]
Olive oil	Purgative	One scoop of olive oil.	[54]
Olive oil	Childhood diarrhea (*sciugghimentu*)	It mentions a traditional mixture made of breast milk, oil, and soot scraped from the kitchen stove hood.	[27]
Olive oil	Constipation	For constipation, boiled water with olive oil is used.	[30]
Olive oil	Constipation	Soups seasoned only with oil.	[30]
Olive oil	Constipation	A teaspoon of olive oil during meals.	[44]
Olive oil	Constipation (children)	Oiled parsley roots were inserted into children’s anuses to lure out worms and facilitate their expulsion.	[34]
Olive oil	Constipation (*stomacu o corpu strittu o stiticu*)	Juice of calamint (*Nepitella*) mixed with olive oil.	[27]
Olive oil	Dyspepsia (*stumacata*, *stomacu* ‘*mmarazzatu*)	As an emetic, one should drink three to four ounces of olive oil.	[27]
**Disease of the skin and subcutaneous tissue**			
Olive oil	Burns	The burned area is anointed with pure olive oil vigorously beaten together with snow.	[52]
Olive oil	Burns	The most severe burns should be covered as soon as possible with oil mixed with snow that fell on a Friday in March and was preserved for special occasions.	[26]
Olive oil	Sunburns	It was moistened with water and beaten oil.	[46]
Olive oil	Burns	Cloths moistened with pure lime water mixed with oil; the preparation of this water required certain precautions…	[33]
Olive oil	Burns	Boil rue, thoroughly crushed, with two parts oil and one part virgin wax, then strain through a linen cloth (ointment of St. Francesca Romana).	[49]
Olive oil	Burns	Apply an ointment prepared with burned walnuts, bay oil, and olive oil.	[49]
Olive oil	Burns	Place some oil in a cup and leave it exposed to the winter air until snow has fallen into it (snow oil), then apply it.	[49]
Olive oil	Burns	Apply to the wound an emulsion prepared by beating olive oil with water.	[44]
Olive oil	Burns	Apply an emulsion prepared by beating egg white with water and olive oil.	[44]
Olive tree	Burns	Reduce seven olive leaves to ash from a tree that has never borne fruit; knead the ashes with sow lard if the patient is female, or with boar lard if the patient is male. Apply the resulting ointment to the affected area.	[49]
Olive	Burns	Wash the affected area with brine (water from olives preserved in salt).	[57]
Olive oil	Burns	Topical applications of melted wax emulsified with olive oil.	[33]
Olive oil	Burns	Anointing with oil and beaten salt.	[33]
Olive oil	Burns	Applications of oil emulsified with a small amount of water.	[33]
Olive oil	Burns	Poultice of scraped or mashed potatoes mixed with flour and sweet oil.	[33]
Olive oil	Burns	Grated potatoes combined with oil and beaten salt.	[33]
Olive oil	Burns	Breadcrumbs, toasted on the oven peel, are kneaded with fine oil; the mixture is then applied to the affected area.	[58]
Olive oil	Burns	The affected area is anointed with pure olive oil.	[59]
Olive oil	Burns	For burns, a wax–oil preparation is used, consisting of wax melted together with olive oil and subsequently allowed to solidify.	[60]
Olive oil	Burns	Burns should be anointed with olive oil.	[61]
Olive oil	Burns	Oil, water, and ink are taken, thoroughly mixed, and used to prepare compresses.	[47]
Olive oil	Burns	If the condition is severe and results in an ulcer, an ointment is prepared, applied with a feather, and the area is bandaged. This ointment can be prepared in various ways, one of which involves mixing oil with salted water and beating thoroughly.	[47]
Olive oil	Burns	For burns, oil beaten together with water is used.	[50]
Olive oil	Burns (oil, water, broth)	Apply oil to the affected area.	[37]
Olive oil	Burns (oil, water, broth)	Take olive oil and a hard-boiled egg yolk, knead them thoroughly, spread the mixture onto a piece of cloth, and then apply it to the affected area.	[37]
Olive oil	Burns (fire)	Take four or five potatoes, grate them thoroughly, and cover the burn for half a day. Then take some oil, a little suet, and some beeswax, prepare a mixture resembling a soft paste, spread it onto a piece of cloth, and apply it to the burn.	[37]
Olive oil	Burns	Oil combined with snow.	[40]
Olive oil	Burns	As a topical remedy, an ointment composed of oil, wax, and egg white, thoroughly blended, is used.	[25]
Olive oil	Burns	In large wounds, olive oil mixed with water is applied.	[34]
Olive oil	Burns	In large wounds, a beaten egg yolk mixed with oil or water is applied.	[34]
Olive oil	Burns (*abbruciatina*, *brusciadina*, *jarsitina*)	Spread old oil over the burned area.	[27]
Olive oil	Boils	Repeated anointing with an ointment prepared from half a carafe of sweet olive oil boiled together with a piece of beeswax.	[33]
Olive oil	Boils	A poultice made of oil, egg yolk, and rye flour was applied.	[34]
Olive oil	Impetigo (*pitìnia*, *putìnia*)	Impetigo is believed to always appear on a Friday, and it is recommended to immediately touch it with the index finger moistened with fasting saliva if one wishes it to disappear by Saturday. The fast must be observed as strictly as if one were to receive communion. In Palermo, even a drop of coffee is forbidden, and the saliva is applied to the impetigo not with the finger but with the underside of the tongue. In Montevago, olive oil follows the application of saliva. In Modica, saliva is combined with some crushed and mixed purcidduzzu di S. Antoni (Oniscus L.). In Mazzara, the saliva must always come from a fasting seventh-born male child. Elsewhere, the effectiveness of the remedy is said to depend not only on the birth order of masculinity but also on the name Settimu or Settimo. In Marsala, all members of the Grassellini family are believed to have the power to heal with saliva. In the Palermo districts of Borgo and Kalsa (inhabited by fishermen and seafaring people), the fasting saliva must come from a man who has crossed the Strait of Messina.	[27]
Olive oil	Calluses	The callus is covered with a piece of paper coated with flour paste; the paper must have a central hole to expose the callus, upon which a drop of burning sulfur, a piece of lard, or boiling oil is applied.	[33]
Olive oil	Skin fissures	Apply oil on top.	[33]
Olive oil	Hand fissures (ragades) (*cripaturi di li manu*, *manu squariati o ciaccati*)	Evening rubs with olive oil and beeswax melted together at home.	[27]
Olive oil	Skin fissures	Spread oil boiled with a small piece of sulfur over the area.	[33]
Olive oil	Ulcers	Turpentine, Greek pitch, red wax, and common oil are combined to make an ointment, which is frequently applied to the wound and then bandaged with scrap paper, resulting in rapid healing.	[35]
Olive oil	Ulcers	Spread egg yolks beaten with oil over the wound.	[34]
Olive oil	Ulcers	Many take an egg white, one ounce of oil, and a little white flour; first, the egg white is beaten well, then the oil is added and beaten again, after which the flour is incorporated to form a paste. This mixture is applied to the wound using a linen cloth, which should be changed periodically. This treatment reduces inflammation and promotes drying.	[47]
Olive oil	Ulcers	Wounds caused by kicks from a horse or mule are treated by applying tow soaked in old oil that has been boiled with sulfur.	[40]
Olive oil	Perniosis	Anoint with oil that has been heated within an onion peel.	[33]
Olive oil	Perniosis	Anoint them with oil in which red chili peppers have been boiled.	[33]
Olive oil	Perniosis	When chilblains bleed, apply an ointment composed of warm oil and camphor.	[33]
Olive oil	Perniosis (*ròsuli*, *mulanca*, *mulànchiari*, *vuzzaredda*, *frascili*, *pedignoni*)	The severe itching caused by chilblains is alleviated with olive oil.	[27]
Oleaster	Oral ulcers	The leaves of the wild-olive tree, which is abundant in Calabria, as well as those of the privet, were held in high regard due to their proven efficacy in treating oral ulcers.	[62]
Oleaster	Oral ulcers	The leaves, when chewed, possess the property of healing oral ulcers.	[32]
Olive tree + formula	Eczema flare	“Lu duveciòre” is an impetiginous eczema that appears in some children around the mouth and chin. To ward it off, three tips of nettle, three of bramble, and three of olive are gathered into a small bundle; this is then immersed in ‘running’ water placed in a dish, and the affected area is sprinkled while reciting a formula.	[26]
Olive oil	Baldness	To promote hair regrowth, take a roof-dwelling tarantula, kill it and dry it in the sun for three days; then boil it in an earthenware pot with oil, which will subsequently be rubbed onto the scalp.	[37]
Olive oil	Dandruff (*canigghiola*)	Olive oil.	[27]
Olive oil	Dandruff (*canigghiola*)	Against dandruff, locally known as caniglia, oil anointments are also employed.	[30]
Olive oil	Cradle cap	Peach leaves are boiled in olive oil; once they have turned yellow, they are discarded, and the oil is used to anoint the child’s head in the evening and morning.	[37]
Olive oil	Cradle cap	Cradle cap in infants should not be disturbed; at most, it may be anointed with a little oil, otherwise it is very likely to become internalized.	[30]
Olive oil	Cradle cap	Olive oil was customarily applied to the affected area	[34]
Olive oil	Cradle cap (impetiginous eczema) (*crusta di latti*, *cunciatura*, *ciaramireddi*)	Bath in olive oil.	[27]
Olive oil	Dermatophytosis/ alopecia	An ointment made of beeswax, oil, rosemary or kerosene, and soot is applied.	[33]
Olive oil	Scrofula	Repeated anointing several times with an ointment made from half a carafe of sweet olive oil boiled together with a piece of beeswax.	[33]
Lamp oil	Adenitis	Its most frequent use is for rheumatic pains, and pains of any other kind; at the onset of erysipelatous and phlegmonous skin inflammations, as well as in adenitis.	[26]
Lamp oil	Fissures	Lamp oil is applied.	[63]
**Diseases of the musculoskeletal system and connective tissue**			
Olive oil	Lumbago (cufi)	Whether the cause is traumatic, rheumatic, neuralgic, etc., the usual old midwife (*commare*), skilled and mysterious, is called into action, and the patient is subjected to a more or less extensive massage, under the pretext of rubbings with the inevitable accompaniment of warm oil, camphorated alcohol, fumigations, and other similar expedients which by now are no longer unknown to anyone.	[64]
Lamp oil	Intercostal pain	When it does not come from pleurisy, it is said to depend on a lowering or falling of the breasts (*zènne*), both in men and in women. There are experts who know how to put them back in place (*a ffarl*’ *aresajje*), first rubbing beneath the breasts with ‘lamp oil,’ and then pushing the ribs strongly upward.	[26]
Lamp oil	Arm and leg pain	If it is winter, a liniment is needed: lamp oil and rosemary are taken and boiled together; when they are well cooked, the painful area is thoroughly anointed with the palm of the hand, and then wrapped in flannel. This should be done two or three times a day, and care must be taken that the oil is always very warm.	[47]
Lamp oil	Rheumatic pain	Its most frequent use is in rheumatic pains and pains of any other kind; at the onset of erysipelatous and phlegmonous inflammations of the skin, as well as in adenitis.	[26]
Olive oil	Rheumatic pain	Rubbings with an ointment made from male incense boiled slowly in olive oil, and then the whole area is wrapped with a woolen cloth.	[33]
Olive oil	Rheumatic pain	Rubbings with chamomile water and beaten (emulsified) oil, and then wrapping the area with a woolen cloth.	[33]
Lamp oil	Phlegmons	Its most frequent use is in rheumatic pains and pains of any other kind; at the onset of erysipelatous and phlegmonous inflammations of the skin, as well as in adenitis.	[26]
Olive oil	Rheumatic congestions in the neck	To dissolve the blockages, rubbings with warm oil and camphor are applied.	[36]
Iron-infused oil	Lumbago	Ointment with iron-infused oil	[33]
Olive oil	Dislocation	When a joint becomes dislocated, the arm or leg is stretched out and rubbed with warm oil. Then the arm or foot is wrapped in a poultice made of bran and vinegar.	[57]
Olive oil	Dislocation/tendinous pain	Rubbings with warm oil.	[33]
Lamp oil	Joint pain	Oil from a lamp burning before the image of Our Lady of Sorrows.	[25]
Olive oil (*ogghiu di Patri Mulè*)	*Rheumatism* (*reuma*, *romaticu*)	Anointings with ‘ogghiu di Patri Mulè,’ an oil in which various aromatic herbs have been cooked and left to infuse, and which is prepared by a Franciscan friar named Father Mulè.	[27]
Olive oil + prayer	*Stiff neck* (*torcicoddu*, *cudduzzu*)	Anointing and rubbing with olive oil while softly repeating a certain prayer which must not be told idly to anyone, and which, only under special circumstances, must be taught on Christmas night.	[27]
Lamp oil	Stiff neck	Take a piece of tow, grease it with warm lamp oil, and apply it to the neck.	[37]
Olive oil + incantation	Stiff neck	People willingly submitted to the charms, fumigations, and rubbings with warm oil that the common folk-healers prepared for them.	[65]
Olive oil	Stiff neck	Iron-infused oil.	[44]
Lamp oil	body pain		[66]
**Diseases of the genitourinary system**			
Olive oil	Kidney pain/retention	To spread warm oil below the navel, accompanied by a poultice of mallow with a teaspoon of saltpeter, lettuce, and ‘aschiuni’ cooked and crushed.	[34]
Olive oil	Female breasts	One pound of common oil, ten heads of garlic boiled together until they become charcoal; then add two ounces of wax, two ounces of rosemary, and one ounce of mastra medolla d’ossa (perhaps the marrow of a bovine femur), melted together, and once removed from the fire, add two ounces of barrel shavings (Rasa de Botte).	[67]
Olive branches + incantation	Sterility	Charms are performed, to tell the truth of little effect, and neither offensive to morals nor harmful to health; and such charms are always accompanied by the fumigation of incense, olive blessed during the Palm Sunday rite, crab eyes, salt, and some other strange substance. The fumigations are made around the sufferer, while the charm is recited in a nasal voice.	[65]
**Pregnancy, childbirth and puerperium**			
Olive tree/incantation	Puerperal fever	Incantations are performed, admittedly of little effect and harmless both to morality and to health, and such incantations are always accompanied by the fumigation of incense, blessed olive from the Palm Sunday rite, crab eyes, salt, and some other curious substances. The fumigations are carried out around the suffering woman while the incantation is recited in a nasal voice.	[65]
Olive tree/incantation	Pregnancy	Incantations are performed, admittedly of little effect and harmless both to morality and to health, and such incantations are always accompanied by the fumigation of incense, blessed olive from the Palm Sunday rite, crab eyes, salt, and some other curious substances. The fumigations are carried out around the suffering woman while the incantation is recited in a nasal voice.	[65]
Oil from the iron lamp	Milk	If a woman, because her child has died or for some other reason, wishes to stop her milk, she has only to anoint her breasts with the oil from the iron lamp.	[26]
Olive oil	Childbirth	For three or four days, the woman in childbed is given mallow and chamomile water, a purgative of olive oil, and after three or four days, some broth and a few slices of toasted bread dipped in wine.	[57]
Olive oil	Childbirth	During childbirth, if the flow of water continues and the delivery threatens to become dry because the fetus does not advance at the same time, the woman should be given broth, wine, and soup with plenty of oil continuously, because these liquids compensate for the lack of water and help the child to slide out more easily; however, at times the amount of wine is increased to the point of causing genuine drunkenness.	[25]
Olive oil	Childbirth	If the woman has difficulty giving birth, she is given water infused with citron seed with some common oil added, or chamomile water again with oil added and as hot as possible, or some cyprus decoction.	[47]
Blessed olive tree	Childbirth	For difficult labor, various items are thrown haphazardly into a small brazier full of glowing embers—leaves of olive blessed on Easter Day, candles from the Ceriola ritual, paper images of saints and Madonnas, chicken feathers, and the husband’s hair—and the laboring woman is fumigated from top to bottom, especially where the thigh turns toward the fullness of the hip.	[66]
Olive oil	Childbirth (womb disorder)	Common oil added to the food porridge.	[47]
Olive oil	Childbirth (swollen belly)	Melted lard, or even ordinary oil, is taken and rubbings are applied over the whole body, and a sheet of sugar paper smeared with lard is left in place on top.	[47]
Olive oil	Nipple fissures (*li serchj*)	Oil with soot.	[43]
Olive oil	Obstructed mammary duc (*U pilu d*’*à minna*)	It causes fever and pain; it is treated with warm oil spread over the breast, and with hot cabbage leaves wilted over the fire applied on top.	[43]
Olive oil	Obstructed mammary duct	Warm olive oil spread over the area where the swelling is greatest, and over the oil, hot cabbage leaves are also covered with ash.	[63]
**Injury, poisonings and certain other consequences of external causes**			
Olive oil	Mushroom poisoning	A large amount of oil is drunk.	[57]
Olive oil	Mushroom poisoning	In case of poisoning, three ounces of oil taken by mouth will make one vomit out everything; and if vomiting does not occur, the oil neutralizes the poison.	[26]
Olive oil	Foreign bodies	Orally, a large quantity of oil is used, as an emetic.	[27]
Lamp oil	Needle or fork punctures under the fingernails	Burn a small piece of cotton and place it over the spot, after first anointing it with lamp oil; this prevents infection from developing in the finger.	[47]
Olive oil	Reptile bites and insect stings velenosi	Apply olive oil to the bite/sting and bandage it immediately and very tightly.	[27]
Olive oil	Drunkenness	Drink a bit of oil.	[68]
Olive oil	Poison	Against vegetable and animal poisons introduced through the stomach, our people have no other general remedies than mechanical emesis: a good glass of olive oil mixed with mallow water, or a bit of theriac. But in some cases of poisoning by plants (aconite, hemlock), they use lamb rennet dissolved in wine.	[25]
Olive oil	Excoriations	If an ulcer develops, beat together one egg white and one ounce of fine olive oil, then apply the mixture to the affected area using a linen cloth; this proves highly beneficial.	[47]
Olive oil	Abrasions (caused by footwear)	Apply the fleshy layers of onion that have been boiled in olive oil.	[33]
Lamp oil + formula	Wounds	Lamp oil was poured onto a piece of cloth, soot scraped from the bottom of the cauldron was dropped onto it, and then it was applied to the wound while reciting a formula.	[33]
Olive oil	Wounds	To staunch bleeding and, above all, to promote cicatrization, one must use oil and soot.	[26]
Olive oil	Wounds	To staunch bleeding and, above all, to promote cicatrization, oil and wine boiled together must be used.	[26]
Iron-infused olive oil	Wounds	To staunch bleeding and, above all, to promote cicatrization, one must use scalding iron-infused oil.	[26]
Iron-infused olive oil	Wounds	Anointment with iron-infused oil (lamp oil in which a red-hot iron has been extinguished).	[33]
Olive oil	Wounds	To staunch bleeding and, above all, to promote cicatrization, one must use oil or St. John’s wine, prepared by infusing aromatic herbs and flowers gathered on the morning of St. John’s Day.	[26]
Sulfurated olive oil (*l’uojje ‘nzulfanate”*)	Wounds	To staunch bleeding and, above all, to promote cicatrization, one must use sulfurated oil (‘l’uojje ’nzulfanate’), prepared by boiling sulfur in oil, of which a few scalding drops are applied to the wound.	[26]
Olive oil	Wounds	Wound bandaging is always performed by local women using strips torn from hemp cloth belonging to a man’s shirt or other male garments, as the use of women’s linen is forbidden—likely due to beliefs regarding the harmful properties of menstrual blood. The strips are then anointed with washed lard or olive oil.	[25]
Olive oil + formula	Wounds	An ointment is prepared from wine and oil (‘vine ’nciarmate’), and a formula is recited. Meanwhile, the ‘nciarmato’ wine is prepared; it is spread on a hemp cloth and applied to the wound. After the wine “ciurmato”, another incantation is recited.	[33]
Olive oil	Wounds	A rag of burnt canvas soaked in oil “cinciazzu” is applied.	[33]
Olive oil	Wounds	The most tender feathers of the heron, or of another bird of the same species, torn from the lower abdomen and moistened in oil are applied to wounds caused by cuts or blows.	[69]
Olive oil	Contusions	The oil kept on the table during Christmas dinner is a very effective remedy against bruises.	[58]
Iron-infused olive oil	Contusions/Bruises	Rubbing with iron-infused oil.	[33]
Olive oil	Contusions/Bruises	When the tumor (swelling) appears, an onion casing filled with crushed charcoal and oil and cooked on the embers is applied.	[33]
Olive oil	Ecchymosis (lividura) contusions (cuntusioni, ammaccatina, pistadina)	In cases of falls from trees, especially walnut trees, bruises are treated with prickly pear leaf juice, with crushed snails, with oil and wax, or with crushed mugwort.	[27]
Olive oil	Lacerations	The oil kept on the table during Christmas dinner is a very effective remedy against lacerations.	[58]
Olive oil	Cuts, lacerations, bruises	For small wounds, cobwebs are used, sometimes dipped in oil.	[34]
Olive oil	Splinters	Shoemaker’s pitch plasters are applied with the addition of oil.	[49]
Olive oil	Splinters	Take the gall of a male pig, dry under the fireplace, grind and mix with oil and then apply it.	[49]
Blessed olive oil	Skin punctures	They were generally cauterized in this way: a hand lamp filled with good olive oil is lit, and a blessed concave olive leaf is filled with the same oil. It is brought to a boil over the flame, and drops are allowed to fall onto the puncture itself. In cases of more serious puncture wounds, or those suspected of being infected because they were caused by rusty tools or stable forks, lemon juice is immediately applied to the wound, while the injured person eats a slice of the lemon.	[70]
Lamp oil	Lymphadenopathy secondary to injury	Rubbings with lamp oil.	[33]
Olive oil	Lymphadenopathy secondary to injury	A tuft of black wool is applied, taken with the tip of a sickle and soaked in oil, aprayer. is repeated	[33]
**Symptoms not elsewhere classified**			
Olive oil	Skin complexion	To obtain a fair (white) face one must look into a bowl of oil on the first Friday of March.	[57]
Olive oil	Childhood fevers (costicelli caduti)	Caused by strain from being constantly carried in the arms. The mothers, already well-versed in all the treatments for children, lay the child face-down across their knees and, with the palm of the hand, rub his ribs and shoulders; and when they believe that he has ‘settled,’ they anoint him with warm oil and wrap him with cabbage leaves warmed and spread with ashes.	[43]
Lamp oil	Various ailments	There is more than one series of oils, dedicated to different saints, parallel to that of the waters, used with pious intention for various ailments.	[26]
Olive tree	Fever	After a few days it is customary to administer a tisane made from tinniruma d’agghiastru, that is, the tender leaves of the common wild olive (oleaster).	[27]
Olive tree	Fever	A decoction of olive leaves, used also as a purifying treatment.	[55]
Olive oil	Fever	Without relying on hopes of magical expedients, anyone who wishes to prevent ‘obstruction’ must eat everything seasoned abundantly with oil.	[26]
Olive tree	Cold fever	Root of olive tree, stripped of its bark and washed, made into a decoction and drunk in the morning on an empty stomach.	[35]
Lamp oil	Fever	Ointment with lamp oil.	[49]
Lamp oil	Children’s chronic illnesses	The oil from the lamp of Saint Vincent is limited to curing children’s chronic illnesses.	[66]
Lamp oil	*Coryza* (*nànfara*, *nànfira*)	Children’s noses are anointed with lamp oil, taking care not to use too much so as not to predispose them to polyps. In Castelbuono these children are put to bed fully dressed.	[27]
Olive oil	Epistaxis (*nasu scugnatu*, *sangu d’ ‘u nasu*, *scugnatina*, *scattatina*, *scattatina r’ ‘o nasu*)	Drink freshly squeezed lemon juice and oil.	[27]
Olive oil	Plantar hyperhidrosis (*suduri di li pedi*, *pedi squariati*, *sudore dei piedi*)	Excessive sweating of the feet caused by long and tiring walking gives rise to the so-called pedi squariati or squadati (‘heated feet’). As a remedy, the feet are anointed with oil, which helps to cool and soothe them.	[27]
Blessed olive oil	All ailments	A true panacea for all ailments is the oil of the Most Holy Sacrament, applied wherever one wishes.	[66]
Lamp oil		“‘Lamp oil’, in folk medicine, is almost a panacea. It simply needs to be taken from a handheld lamp—usually made of iron—while it is lit.	[26]
Lamp oil/iron-infused oil		‘Lamp oil’, in folk medicine, is almost a panacea. It is even better if taken from the lamp burning before a sacred image; when a stronger action of the lamp oil is desired, a red-hot iron must be extinguished in it.	[26]
**Magical/ritual use**			
Olive tree	Evil eye (*affàscinu*)	Woe to anyone who leaves baby bands and infants’ linen out to dry after sunset! The evil spirits, which begin to wander about precisely at dusk, would impart to those garments sinister powers that would harm the health of the little, innocent creatures—so delicate, and therefore all the more exposed to their effects. A newborn who wore such clothes would become dissumbrata (‘overshadowed’) and would find no rest. Thus, the careful mother, or the good, attentive grandmother, if by chance they had fallen into such negligence, would never use that linen or those bands without first thoroughly fumigating them with blessed olive branches burned over glowing embers.	[50]
Olive oil	Bewitchment	To determine whether the fever or headache affecting a child is caused by the evil eye or not, a drop of oil is let fall into a basin filled with water. If the oil remains floating on the surface, there is no evil eye; if it disappears, then yes, the evil eye is present.	[26]
Blessed olive oil	Bewitchment	Very common among the peasants of Friuli are certain triangular pouches, made by folding diagonally one of the two squares of the scapular. Into these pouches they place a few drops of wax from the Easter candle and from the triangolo candle, a grain of incense and one of myrrh, a leaf of blessed olive, and a grain of blessed salt—the kind used in baptism. And, if it can be obtained, a bit of cotton wool dipped in holy oil, and perhaps even a tiny fragment of a consecrated host. The pouch is then sprinkled with holy water and worn around the neck as protection against magic spells. Pouches in the shape of a heart, prepared in the same manner, are also in use.	[34]
Olive oil	Evil eye	Half a glass of water is poured into a basin, and three drops of lamp oil are allowed to fall into it. On top of the drops three grains of wheat taken from an ear gathered in the mountains at least three years earlier are placed, and a certain phrase is then recited. Finally, the basin is covered with a sieve, and an overturned pan was set on top of it; at the slightest movement of the pan, the evil eye was considered to have disappeared.	[33]
Olive oil	Evil eye	A mixture was prepared from holy water, wine, and oil; a sprig of blessed olive was dipped into it, and with this the child was sprinkled while secret prayers were recited.	[69]
Olive tree	Evil eye	A kind of amulet, called *giesu*, was placed among the infant’s swaddling bands. It was a small heart-shaped pouch containing a pinch of salt, some incense, blessed olive leaves, and blessed palm leaves.	[71]
Olive tree	Evil eye	Incense, dried olive leaves, and salt were scattered onto the fire; the child was held over the smoke, and the wise-woman, tracing crosses, repeated certain words…	[31]
Olive oil	Evil eye	Curious is the method used to detect and remove the evil eye. It consists of pouring three drops of olive oil into a plate of clear water while saying: ‘Others have given it to you; I take it away from you.’ If the drops float and remain together, there is no evil eye; if the drops drift apart and spread out, then the evil eye was present but has been removed.	[58]
Olive tree	Evil eye	A charm was prepared with two grains of wheat, two grains of salt, and two blessed olive leaves. Everything was placed in a small pouch to be worn close to the heart for 10 days. After that, the pouch was burned. Ten days later, the person who had cast the evil eye would come asking for some salt, which you had to refuse, thus forcing them to ask for forgiveness.	[38]
Olive tree	Evil eye	A mixture made of holy water, wine, and oil was prepared. A blessed olive twig was dipped into it, and the child was sprinkled with it while secret prayers were recited.	[69]
Olive tree	Children’s evil eye	A small pouch was used, containing various ingredients:	[30]
		(a) three heads of *poste re ciucce* (nails used for shoeing donkeys) shaped by the blacksmith, three pinches of salt, three grains of wheat, and three black chickpeas;	
		(b) three needle tips arranged *in cross* and attached to images of saints, a small devotional booklet; three grains of wheat from three different granaries, three small stones taken from a crossroads, and three grains of salt;	
		(c) three olive leaves, a piece of stole, a piece of chasuble, a *camuzze re cannela llu seburche* (a small candle stub from the Holy Sepulchre of Holy Thursday), and the usual three heads of donkey-shoe nails;	
		(d) a small piece of badger skin with hair, stones or small stalactites from the Sanctuary of Novi, and some old coins (*the bel carniedde*: a “carlino”, for example).	
Olive tree	Spells	To protect oneself against spells, one had to wear under the shirt a small amulet made with pieces of fishing nets, containing a little pouch of sand, seven small nails, three olive leaves, and three grains of salt.	[72]
**Cosmetic use**			
Olive oil	Hair	Applications of olive oil in which a live lizard is thrown into the oil while boiling.	[56]
Olive oil	Hair	Olive oil is applied, which strengthens the hair roots and keeps the scalp free from dandruff, a major cause of hair damage.	[37]
Olive oil	Hair	To soften the hair, rinse the hair with a tablespoon of oil beaten into one liter of water.	[44]

**Table 2:** Ethnobotanical knowledge and contemporary uses of *Olea europaea* L. in Italy recorded at the beginning of the third millennium through surveys and semi-structured interviews.

Remedy	Pathology	Administration	Reference
**Certain infectious and parasitic diseases**			
Oil	Intestinal worms	Eaten raw *Allium sativum* L. with olive oil against intestinal worm.	[78]
Oil	Antihelmintic	*Tanacetum vulgare* flower head macerated in olive oil.	[79]
Oil	Antihelmintic	With *Ruta graveolens* leaves to massage as antihelmintic.	[80]
Oil	Against intestinal worms	Olive oil together with a *Allium sativum* L. clove is used to make an ointment useful against intestinal worms.	[81]
Leaves	Against malaria	In order to prevent malaria fever attacks, patients were advised to drink an infusion of willow and olive leaves every morning.	[82]
Oil	Fuoco di Sant’Antonio (Herpes zoster)	*Lilium candidum* in the form of a poultice prepared by frying the bulb in olive oil and applying this externally against shingles.	[83]
Oil	Against tinea	A maceration of *Rosamarinus officinalis* L. in olive oil is rubbed on the scalp against tinea.	[84]
Oil	Herpes labialis	To heal labial herpes.	[85]
Oil	Anti-lice	Anti-lice (children).	[85]
Oil (warm)	Mumps	Red-hot coal-shovel or tongs were immersed in the oil to prepare “olio ferrato” with fried oil. This was applied to swollen ears with cotton wool and kept in place with a woolen scarf.	[86]
Oil	Mumps	Variant with chamomile added and a poultice of warm oil placed on the ear.	[86]
Oil (fried)	Persistant earache from mumps or infection	A few drops in the ear.	[86]
Oil	Cataplasm	Anti-inflammatory. Cataplasms of fresh leaves of *Nicotiana tabacum* L., rapidly fried in olive oil, are applied locally and wrapped in warm cloths to treat parotitis.	[87]
**Endocrine, nutritional and metabolic diseases**			
Leaves	Diabetes mellitus	Decoction.	[88]
Oil	Hypercholesterolemia	Oral.	[89]
Leaves	Hypoglycemic	Alcohol and water macerate.	[78]
Leaves	Hypercholesterolemia	Decoction.	[88]
**Diseases of the nervous system**			
Oil	Sciatica	Ruta graveolens leaves fried in olive oil massaged against sciatica.	[90]
Oil	Aching nerves	Ointment made of *Hypericum perforatum* L. flowers macerate in olive or linen oil to spread on aching nerves.	[91]
Oil	Neuralgia	‘‘Oil of chamomile’’ obtained by macerating the dried heads of *Matricaria chamomilla* L. in oil olive is applied to painful parts in cases of neuralgia.	[84]
Oil	Neuralgia	Leaf of *Ruta graveolens* L. macerated in olive oil is used in cases of neuralgia.	[81]
Oil	Sciatica	*Ruta chapensis* L. young shoots infused in olive oil applied to areas suffering from sciatica.	[89]
Oil	Headache	Fresh leaves of *Clematis vitalba*, crushed or steeped in alcohol or olive oil, are applied to treat headache.	[92]
Oil	Headache	By massaging warm oil with leaves of *Ruta chalepensis*.	[78]
**Diseases of the eye and adnexa**			
Oil	Ophtalmic disinfectant	*Verbascum chaixii* flowers macerated in olive oil.	[79]
Oil	Stye	Olive oil is put on stye to heal it quickly.	[81]
Oil	Eye anti-inflammatory	An olive oil macerate leaves of *Ruta graveolens* L. is applied topically as an eye anti-inflammatory.	[93]
**Disease of the ear and mastoid process**			
Oil	Earache	Warm oil is introduced into the ear as an earache reliever.	[94]
Oil	Earache	Warm oil mixed with *Prunus Persica* L. kernil as an earache reliever.	[94]
Oil	Otitis	Ear drops for otitis.	[95]
Oil	Otitis	Chamomile left to steep in olive oil is applied topically as an analgesic for otitis.	[92]
Oil	Otitis	Warmed olive oil with Petroselinum crispum for otitis.	[95]
Oil	Ear-ache	Rubbed onto ears.	[86]
Oil (warm)	Earache	Red-hot coal-shovel or tongs were immersed in the oil to prepare “olio ferrato” with fried oil. This was applied to ears with cotton wool and kept in place with a woolen scarf.	[86]
Oil	Ear pains	Topical use of the warm oil.	[96]
Oil	Anti-otalgic	Instillation of hot oil.	[83]
Oil	Non-specific	As an emollient, it was also quite common to place a few drops of olive oil into the ear to remove excessive earwax.	[82]
Oil	Otitis		[97]
Oil	Analgesic/Anti-inflammatory	Some drops of warm oil are poured inside the ear in cases of otitis.	[87]
Oil	Analgesic/Anti-inflammatory	Boiled onions, mashed and added with olive are applied locally in cases of otitis.	[87]
Oil	Earache	Oil heated and put on the ear lobe.	[91]
Oil	Inflammation	*Laurus nobilis* L. fruits fried in olive oil are used in the topical treatment of auricular inflammations.	[98]
Oil	Earache	Hot oil (heated in halfeggshell on embers) to heal earache.	[99]
Oil	Earache	Leaf juice of *Ruta graveolens* heated together with a bit of olive oil, is placed into ears in case of otitis or ear infections to hill and reduce earache.	[81]
Oil	Earache	Flower and leaf juice of *Malva sylvestris* L. warmed in olive oil is useful in case of earache.	[81]
Oil	Ear infections	Cloves of *Allium sativum* L. are crushed and mixed with olive oil to obtain an ointment used to treat ear infections.	[81]
Oil	Othorrea	The decoction of *Ruta chalepensis* fresh leaves in olive oil is poured warm in the ear.	[100]
Oil	Earache	Oil (compress) for earache.	[101]
Oil	Ear infections	Olive oil cures ear infections.	[81]
Oil	Otitis	Drops of warm oil are placed in the ear to treat otitis.	[92]
Oil	Otitis	Cotton wool soaked in oil is steam-heated and placed in the ear with otitis.	[92]
Oil	Otitis	Olive oil soothes earache caused by otitis.	[81]
Oil	Earache	With *Cyclamen hederifolium* Aiton bulbs.	[80]
Oil	Otitis	Topical use.	[102]
Oil	Otitis	Two or three drops of warm oil in the ear to cure otitis.	[89]
Oil	Ear pains	By massaging warm oil with leaves of *Ruta chalepensis.*	[78]
**Diseases of the circulatory system**			
Leaves	Hypertension	Infusion in water two or three times a day.	[94]
Leaves	Hypertension	Decoction or macerated in water.	[95]
Branches	Hypertension	The decoction of boiled tender branches is drunk in the morning on an empty stomach.	[82]
Leaves	High blood pressure (as diuretic)	A glass drunk early morning on an empty stomach of the decoction made from three cloves (*strusci*) of garlic and seven olive leaves. Alternatively, a decoction of merely the leaves (“it’s very bitter, but does you good”).	[86]
Leaves	Hypertension	Decoction.	[103]
Leaves	Hypertension	Several leaves in decoction to cure hypertension.	[104]
Leaves	Hypotensive	Decoction to treat hypertension.	[105]
Leaves	Hypotensive	Hypotensive.	[106]
Leaves	Hypotensive	Decoction to treat hypertension.	[107]
Leaves	Hypertension	Decoction.	[108]
Leaves	Hypertension	Leaves decoction as hypotensive.	[109]
Leaves	Hypertension	Decoction.	[96]
Leaves	Hypertension	Decoction of the leaves claimed to have hypotensive properties.	[90]
Oil	Hypertension		[97]
Leaves	Antihypertensive	Infusion.	[79]
Leaves	Hypertension	Decoction.	[88]
Leaves	Circulatory system diseases		[110]
Leaves	Hypertension	Leaf decoction used as hypotensive.	[111]
Fresh leaves	Hypertension	A maceration is claimed to be effective in case of high blood pressure.	[98]
Leaves	Hypertension	Chewing the leaves has a hypotensive effect.	[84]
Leaves	Hypertension	Leaf decoction as hypotensive.	[99]
Leaves	Hypertension	A decoction of the leaves promotes diuresis.	[84]
Leaves	Hypertension	A decoction of olive leaves is drunk as an antihypertensive.	[92]
Leaves	Hypertension	A decoction of fresh leaves is considered anti-hypertensive.	[112]
Leaves	Hypertension	A water macerate is used as a hypotensive.	[93]
Oil	Circulation/chilblains	Leaf juice of *Urtica dioica* L. mixed with olive oil (with or without salt) is used as a lotion able to activate circulation and is useful against chilblains.	[81]
Leaves	Hypertension	Infusion for controlling high blood pressure.	[113]
Oil	Hypertension	Cloves of *Allium sativum* L. are crushed and mixed with olive oil to obtain an ointment applied under the feet soles to lower the blood pressure.	[81]
Leaves	Hypertension	A decoction is used to low the blood pressure.	[114]
Leaves	Hypertension	Decoction (to drink) as hypotensive.	[81]
Leaves	Hypertension	A decoction (100 leaves boiled for 30 min and filtered, or 20 leaves boiled for 15 min) is taken orally (a small glass a day for 7/10 days).	[115]
Leaves	Hypertension	Decoction.	[80]
Leaves	Hypertension	Decoction.	[89]
Leaves	Hypertension	Infusion.	[116]
Leaves	Hypertension	Decoction: alcohol and water macerate.	[78]
Oil	Anti-hemorrhoids	Ointment made by mixing the decoction of *Aesculus hippocastanum* L. bark and leaves with olive oil or pig fat against hemorrhoids.	[85]
Oil	Hemorrhoids	*Verbascum chaixii* flowers macerated in olive oil	[79]
Leaves	Hemorrhoids	A decoction of the leaves acts against piles.	[117]
Oil	Hemorrhoids	With *Cyclamen hederifolium* Aiton bulbs.	[80]
Seeds/oil	Hemorrhoids	The seeds are pounded, mixed with olive oil, wrapped in a gauze and applied locally as a poultice.	[115]
Oil	Hemorrhoids		[97]
**Diseases of the respiratory system**			
Oil (warm)	Sore throat	Red-hot coal-shovel or tongs were immersed in the oil to prepare “olio ferrato” with fried oil. This was applied with cotton wool and kept in place with a woolen scarf.	[86]
Leaves	Common cold	Inhalation.	[108]
Oil	Sore throats	A towel soaked with oil to treat sore throats.	[118]
Oil	Bronchitis	Warm oiled paper applied to chest against bronchitis.	[111]
Oil	Bronchitis	Warm oil massaged on the chest for pectoral affections like bronchitis.	[90]
Leaves	Decongestant	Packs of leaves boiled in water on chest as decongestant.	[99]
Oil	Bronchitis	Hot oil for rubbing on chest against bronchitis.	[99]
Oil	Troatache	Topical use of oil.	[78]
Oil	Cold/bronchitis	Topical use of oil on nose and breast.	[78]
Oil	Throat inflammation	By massaging oil on the neck, sometimes with leaves of *Rumex* spp.	[78]
Oil	Tonsillitis	A towel soaked with oil on the throat or by massaging oil on the wrist against tonsillitis.	[89]
**Diseases of the digestive system**			
Leaves	Appendicitis	Fresh leaves chewed.	[95]
Oil	Non specified	Tablespoon of oil as liver depurative.	[95]
Oil	Laxative	The boiled leaves of *Crepis capillaris*, seasoned with olive oil, are eaten to induce intestinal function.	[92]
Oil	Laxative	Olive oil has a laxative effect if taken on an empty stomach.	[82]
Gum	Toothache	Application of gum (from branches).	[103]
Oil (extract)	Laxative	Used to treat constipation.	[106]
Leaves	Intestinal stimulant	Inhalation.	[108]
Raw fruits/oil	Mild laxative	Cooked raw fruits and oil.	[83]
Leaves	Stomachache	Chewed.	[119]
Aerial part	Hepatoprotective	To provide hepatoprotection.	[120]
Aerial part	Stomachache	Against stomachaches.	[120]
Oil	Constipation	One spoon of oil drunk before eating, against constipation.	[90]
Oil	Laxative		[97]
Oil	Spasmolytic	Lukewarm olive oil is used in abdominal massages against intestinal spasms, especially for babies.	[87]
Oil	Spasmolytic	Fresh leaves of *Cynoglossum creticum*, warmed up and smeared with olive oil, are applied externally to relieve abdominal pains.	[87]
Oil	Spasmolytic/analgesic	In cases of abdominal pains and intestinal spasms fresh leaves of *Nicotiana tabacum* L., warmed up and smeared with olive oil, are applied locally and kept on in bandages till the pain is relieved.	[87]
Oil	Spasmolytic	Lukewarm olive oil is used in abdominal massages against intestinal spasms, especially for babies.	[87]
Leaves	Stomachic	Decoction (with *Cynodon Dachylon*, *Tussilago farfara* and *Equisetum arvense*).	[88]
Oil	Intoxication due to alcohol: raw (eaten with vinegar and olive oil)	Raw leaves of *Sonchus arvensia* L. eaten with vinegar and olive oil.	[88]
Oil	Constipation	Olive oil used against constipation.	[111]
Leaves	Stomach acidity	Chewing the leaves claimed to lower stomach acidity.	[84]
Leaves	Wheting liver	A decoction of the leaves whets liver.	[84]
Leaves	Hepatic functions or gallstones	Olive leaf decoction used for its beneficial effects on liver, or to treat gallstones.	[121]
Oil	Hepatic functions	The oil taken on an empty stomach promotes hepatic functions.	[84]
Leaves	Detox	Infusion.	[122]
Oil	Anti-spasmodic	*Diplotaxis tenuifolia* (L.) DC. cooked leaves are eaten with olive oil and lemon juice as an antispasmodic in case of colic.	[93]
Oil	Abdominal pains	By massaging warm oil with leaves of Ruta chalepensis.	[78]
Oil	Toothache	A cataplasm prepared with boiled leaves of Verbascum pulverulentum and smeared with olive oil is used for dental pains.	[100]
Oil	Antispasmodic	*Allium cepa* L bulbs boiled with the bulbs of *Allium sativum* and olive oil as antispasmodic.	[78]
Oil	Laxative	*Petroselinum crispum* (Mill.) Fuss dipped in olive oil and used to tickle the children anuses.	[78]
Leaves	Stomach remedy	Leaves chewed.	[123]
Oil	Colics	*Rumex alpinus* fresh leaves sprinkled with olive oil or honey (poultice) as remedy for intestinal colics.	[124]
Oil	Constipation	Oral	[89]
Oil	Toothache	*Ruta chapensis* L. young shoots infused in olive oil against toothache.	[89]
Oil	Toothache	An ointment made by *Triticum aestivum* L., boiling water and olive oil locally used against toothache.	[80]
Leaves	Jaundice	Jaundice is known locally as su male ‘e s’istria (which means ‘barn owl’s disease’), because the coming of a barn owl is believed to portend this illness. Four different plants (*Artemisia arborescens*, *Helychrysum italicum,* blessed olive and palm tree leaves), together with salt and holy water, are involved in this complicated rite, which must be performed by a healer and repeated for three days.	[115]
Oil	Constipation	The leaves of *Beta vulgaris* L., boiled with Malva sylvestris leaves and dressed with olive oil, vinegar and salt, are taken as food.	[115]
Oil	Drunkness	Half a glass of oil drunk is recommended to neutralize the symptoms of drunkenness.	[92]
Olive	Drunkness	Olives are purifying, so they are eaten before drinking alcohol.	[81]
Oil	Gallstones	Two spoons of olive oil are helpful against gallstones.	[81]
Oil	Stomach swelling	Two spoons of olive oil are helpful against stomach swelling.	[81]
Oil	Constipation	One teaspoon of olive oil a day on an empty stomach promotes intestinal transit.	[81]
Oil	Digestive	Cloves of *Allium sativum* L. are crushed and mixed with olive oil to obtain an ointment used topically on the belly for digestive issues	[81]
Oil	Laxative	Flower infusion of *Matricaria chamomilla* L. asumed with a drop of olive oil has a laxative effect	[81]
Oil	Toothache	A ball is made with leaf pulp of *Petroselinum crispum* (Mill.) Fuss together with salt and olive oil and used against toothache.	[81]
Leaves	Liver stones	A glass drunk early morning on an empty stomach of a decoction made from three cloves (‘strusci’) of garlic and seven olive leaves. Alternatively, a decoction of merely the leaves can be used (“it’s very bitter, but does you good”).	[86]
**Diseases of the skin and subcutaneous tissue**			
Oil	Emollient/burns	The “washed” oil, that is, emulsified with water, is applied topically to burns as an emollient. Pure oil, oil and flour, oil and salt, as well as oil, water, and lemon, are used for the same purpose.	[94]
Oil	Emollient	Oil, water, and lemon, is also used for hands reddened by the cold.	[94]
Oil	Emollient/lenitive	For cracks (*crepoli*), or chapped hands often caused by the cold, a cream made from oil and beeswax is used.	[94]
Oil	Emollient	Ointment applied to calluses, burns, and skin redness in children.	[95]
Oil	Rashes	For rashes of the legs, oil is mixed with water to produce a kind of cream.	[66]
Oil	Burns	Oil alone or emulsified in water is applied to burns	[92]
Oil	Wounds	Oil together with sieved ashes is used as a cicatrizant for wounds.	[92]
Oil	Burns	Oil was mixed with bread crumbs and put on the burns.	[104]
Oil (extract)	Baldness	Used as baldness emollient.	[106]
Oil (extract)	Erythema	Used to treat erythema.	[106]
Oil	Burns	Oil is applied on the burns.	[107]
Oil	Pimples	The poultice of *Rumex crispus* leaves with oil is used to cure pimples.	[114]
Oil	Warts	Topical use of the cream obtained with oil and melted wax.	[96]
Oil	Weak hair	Topical use of a cream obtained with fruit juice of *Citrus* x limon and albumen.	[96]
Oil	Wounds	Poultice of *Scrophularia trifoliata* fresh leaves with olive oil is used as a cicatrizant.	[100]
Oil	Acne	Topical use of the oil with fruit of *Solanum lycopersicum.*	[96]
Oil	Anti-inflammatory/cicatrizing	The crushed leaves of *Malva sylvestris* mixed with olive oil are applied to wounds for anti-inflammatory and cicatrizant purposes.	[92]
Oil	Bruises	Topical use of the oil by massaging.	[96]
Oil	Dry skin	Topical use, sometimes with fruit juice of *Citrus* x *limon* and yolk.	[96]
Oil	Vulnerary	External application of oil was used against burns.	[83]
Oil	Antiseptic	Oil and vinegar mixed together to make an antiseptic “cream”.	[90]
Oil	Analgesic/Anti-inflammatory	Maceration of *Hypericum perforatum* aerial part in olive oil in the sun light during 40 days. Massaged on swellings and burns.	[90]
Oil	Boils/furuncles/abscesses	After washing, fresh leaves of *Rubus ulmifolius* Schott were fixed on the skin with a bit olive oil to mature boils and furuncles/abscesses.	[90]
Oil	Skin disease/vulnerary		[97]
Oil	Anti-inflammatory	Resin of *Larix decidua* with olive oil and beeswax.	[125]
Oil	Lenitive/antiseptic	Olive oil is used as lenitive, soothing, cicatrizant. Oil is applied externally for burns, abrasions, sunrash (olive oil is often used as an excipient for cataplasms (*Allium cepa*, *Asphodelus microcarpus*, *Cynoglossum creticum*, *Malva parviflora*, *Nicotiana tabacum*, *Rumex* spp.).	[87]
Oil	Cicatrizing	*Angelica sylvestria* flowers and leaves macerated in olive oil.	[79]
Oil	Lenitive/antiseptic	Fresh leaves of *Nicotiana tabacum* L. smeared with olive oil are applied and wrapped in gauzes to treat dermatitis and eczemas.	[87]
Oil	Resolvent/anti-inflammatory	Boiled and drained leaves of *Malva parviflora* are pounded with olive oil and applied on sebaceous cysts or furuncles.	[87]
Leaves	Skin diseases		[110]
Oil	Burns	The fresh flower tips of *Hypericum perforatum*, put in olive oil and left to steep in the sun in a sealed jar for about 20 days is considered to be excellent for local treatment of burns.	[92]
Oil	Burns	Applications.	[111]
Oil	Burns/sunburns/skyn erythema	Ointment made of *Hypericum perforatum* L. flowers macerate in olive or linen oil to spread on burns, on sunburn, on skin erythema.	[71]
Oil	Dry and chapped skin	Locally rubbed it acts a protective and restructuring emollient for dry and chapped skin.	[84]
Oil	Strengthens hair	In compresses on the scalp strengthens hair.	[84]
Oil	Strengthens nails	Mixed with drops of *Citrus limon* (L.) Osbeck strengthens nails.	[84]
Oil	Burns/scars	An ointment is prepared by boiling oil mixed with beeswax; this must be cooled in water, then the first three layers are removed and the remainder is stored in a hermetically sealed container to use on burns in order to avoid residual scars	[84]
Oil	Bruises and swelling	An ointment obtained by macerating *Hedera helix* L. bark and fresh leaves in olive oil is applied to bruises and swelling.	[84]
Oil	Lenitive	The decoction of the *Sambucus nigra* L. green bark, boiled in olive oil, is applied to burns as lenitive.	[84]
Oil	Burns/sunburns/rash	‘‘Oil of chamomile’’ obtained by macerating the dried heads of *Matricaria chamomilla* L. in olive oil is effective on burns and against sunburn or rash.	[84]
Oil	Burns	The petals of *Rosa canina* L. macerated in olive oil speed up the healing of burns.	[84]
Bark	Wounds	To help healing wounds.	[121]
Oil	Prevents scarring after burns	An ointment from olive oil, prepared and used according to careful instructions, prevents scarring after burns.	[84]
Oil	Burns	Oil to heal burns.	[99]
Oil	Calluses	Hot oil to heal calluses.	[99]
Oil	Calluses	*Allium sativum* L. bulb poultice with olive oil or beeswax to heal calluses.	[99]
Oil	Corns/warts	A poultice of the crushed fresh leaves of *Sempervivum arachnoideum*, mixed with olive oil is used to remove corns, warts and is used to cicatrize sores and bites.	[126]
Oil	Emollient	Ointment with olive oil and flowers of *Calendula officinalis* L. used as emollient.	[99]
Oil	Burns	Flowers of *Hypericum perforatum* L. in olive oil, then put in the sun, as cicatrizer, against burns.	[99]
Oil	Burns	Ointment with other taxa to treat burns.	[122]
Oil	Burns	Aerial part of *Hypericum perforatum* L. macerated in olive oil to treat burns.	[122]
Oil	Anti-inflammatory	A cataplasm prepared with boiled leaves of *Verbascum pulverulentum* and smeared with olive oil is used as a softening agent in local skin inflammations.	[100]
Oil	Burns	Flowers of *Hypericum perforatum* L. preserved in olive oil and locally applied to treat skin burns.	[122]
Oil	As cicatrizer	Bark of *Sambucus nigra* L. mixed with olive oil or beeswax; it is claimed to act as a cicatrizer.	[93]
Oil	Lenitive/cicatrizing	*Hypericum perforatum* L. crushed fresh plants or an olive oil macerate were used as a lenitive and cicatrizer.	[93]
Oil	As dry hair nourishment	Topical use of oil.	[78]
Oil	Emollient: burn blisters	Topical use of oil.	[78]
Oil	Bruises	By massaging warm oil with leaves of *Ruta chalepensis.*	[78]
Oil	Acne/pimples/lumps	Topical use of oil with flour of *Triticum aestivum.*	[78]
Oil	Burns	Topical use.	[89]
Oil	Burns	*Dryopteris filix-mas* aerial parts cooked in olive oil, are used as a topical lenitive.	[127]
Oil	Burns	*Hypericum perforatum* L. whole plant macerated in olive oil against skin burns.	[89]
Oil	Sunburns	*Solanum lycopersicum* L. fruits with olive oil to treat sunburns.	[89]
Oil	Emollient	Emollient for the skin.	[73]
Oil	Anti-inflammatory	Skin toner in case of inflammation caused by adverse atmospheric events or in babies.	[85]
Oil	Burns	Mixed with water; works against burns.	[85]
Oil	Anti-inflammatory	Mixed with water to relieve anal inflammation in babies.	[85]
Oil	Pimples	Topical use.	[102]
Oil	Burns/sunrash	Topical use.	[102]
Oil	Burns	Locally applied to heal burns.	[80]
Oil	Burns	With *Hypericum perforatum* flowers.	[80]
Oil	Skin rushes	The flowering shoots of *Hypericum perforatum* L. are macerated in olive oil, in sunlight, for 40 days; the liquid is filtered and applied locally.	[115]
Oil	Sunburns	An emulsion of olive oil and water is applied locally as a lenitive.	[115]
Oil	Burns/sunburns	Scalds, burns, solar erythema, wounds: the ‘oleolito’ (flowering shoots soaked in olive oil in sunlight for 20–40 days, then filtered) is applied locally as a lenitive and cicatrizant.	[115]
Oil	Burns	Used to treat every burn.	[121]
Oil	Burns	Olive oil is applied on burns to prevent blister formation.	[81]
Oil	Wounds	*Clematis vitalba* leaves chewed with olive oil and then applied on the wounds.	[113]
Oil	Burns	*Hypericum perforatum* aerial parts preserved in olive oil for skin (especially burns).	[113]
Oil	Burns	*Sambucus nigra* L. bark is boiled in olive oil to obtain an ointment useful against burns.	[81]
Leaves	Burns/dry skin	Leaves together with *Sambucus nigra* L. are used to make an oleolite useful against burns and dry skin.	[81]
Oil	Emollient	Leaves and flowers of *Borago officinalis* L. oleolite (in olive oil) is used for dry skin.	[81]
Oil	Emollient	The plant of *Hylotelephium maximum* (L.) Holub combined with beeswax, olive oil, and a few sprigs of *Sambucus nigra* L. is used to make a regenerating and healing cream for chapped skin, especially for winter rhagades.	[81]
Oil	Used as aftersun	This oleolite is prepared with flowers of *Hypericum perforatum* L. in 100 mL of olive oil (or vaseline oil), then it is left under the sun for 2 weeks, shaking it from time to time. The use of this oil is not recommended before sun exposure since it might induce black spots on the skin; on the contrary, it is useful as aftersun.	[81]
Oil	Burns/shingles	Flower and leaf juice of *Malva sylvestris* L. mixed with olive oil is used to treat burns and shingles.	[81]
Oil	Burns	An ointment made with *Sambucus nigra* L. and Ulmus minor Mill. bark together with olive oil is used in case of burns.	[81]
Oil	Burns/skin diseases	An ointment made with the bark of *Ulmus minor* Mill. and olive oil is used on burns and to treat skin diseases.	[81]
**Diseases of the musculoskeletal system and connective tissue**			
Oil	Against muscular pain	Olive oil macerate of *Juniperus communis* against muscular pains.	[83]
Oil	Against muscular pain	Aerial parts of *Ruta graveolens* is fried in olive oil in topical applications against muscular pains.	[119]
Oil	Against muscular pain	Leaves of *Diplotaxis tenuifolia* fried in olive oil are applied topically to heal muscular pains (especially in the shoulder region).	[119]
Oil	Anti-rheumatic	Arnica montana flower head macerated in olive oil.	[79]
Oil	Anti-rheumatic	*Tanacetum vulgare* flower head macerated in olive oil.	[79]
Oil	Muscular pain	Fresh leaves of *Clematis vitalba*, crushed or steeped in alcohol or olive oil, are applied to joint or muscle pains.	[92]
Oil	Rheumatic pains	The leaves of *Ruta graveolens* simmered in olive oil are used in massages for joints affected by rheumatic pains.	[92]
Oil	Muscular pain	Massaged on any part of the body against muscular pain.	[90]
Oil	Muscular pain	*Ruta graveolens* leaves fried in olive oil massaged against muscular pain.	[90]
Oil	Rheumatisms, arthritis, muscle pains	*Arnica montana* macerate in olive oil, externally applied.	[128]
Oil	Rheumatism	*Rumex alpinus* fresh leaves sprinkled with olive oil or honey (poultice) as remedy for rheumatisms and sprains.	[92]
Oil	Rheumatism	*Ruta graveolens* leaves fried in olive oil massaged against rheumatism.	[90]
Oil	Analgesic/Anti-inflammatory	Maceration of *Hypericum perforatum* aerial part in olive oil in the sun light during 40 days. Massaged to heal pains, rheumatisms.	[90]
Leaves	Gout	A decoction of the leaves acts against gout.	[117]
Oil	Anti-myalgic/analgesic	Fresh leaves of *Cynoglossum creticum*, warmed up and smeared with olive oil, are applied externally to relieve intercostals pains.	[87]
Oil	Anti-rheumatic	Pounded roots, fresh or boiled of *Asphodelus microcarpus* are added with olive oil are applied on joints to relieve rheumatic pains.	[87]
Oil	Rheumatism	Poultice of *Scrophularia trifoliata* fresh leaves with olive oil is used as a cicatrizant.	[100]
Oil	Arthritis	*Hypericum perforatum* aerial parts preserved in olive oil for arthrosis.	[113]
Oil	Antirheumatic	The leaves of *Ruta graveolens* L. and fruits of *Capsicum annum* L. are fried in olive oil; this preparation is applied topically as an antirheumatic.	[98]
Oil	Arthritis/rheumatic	A cataplasm with *Clematis vitalba* L. leaves scalded in olive oil is rubbed on painful joints in cases of arthritis and rheumatic pains.	[84]
Oil	Rheumatic pain	A cataplasm prepared with boiled leaves of *Verbascum pulverulentum* and smeared with olive oil is used for rheumatic pains.	[100]
Oil	Rheumatic pain	Against rheumatic pain.	[99]
Oil	Anti-rheumatic	Oleolite derived from the plant of *Ruta graveolens* fried in olive oil and applied topically.	[129]
Oil	Fracture	Dried and minced galls of *Rhododendron ferrugineum* (plaster with olive oil) as little fracture remedy.	[124]
Oil	Muscular or joint pains	Leaf of *Ruta graveolens* L. macerated in olive oil is used in cases of muscular or joint pain.	[81]
Oil	Gout	Oleolite derived from dried Sambucus nigra flowers fried in olive oil.	[129]
Oil	Inflammation	Aerial part of *Ajuga reptans* L. oleolite (in olive oil) is used for inflamed joints.	[81]
Oil	Muscular pain	Topical use.	[102]
Oil	Anti-rheumatic	Oleolite derived from the plant of *Solanum nigrum* fried in olive oil and topically applied.	[129]
Oil	Arthritis	*Parietaria judaica* L. aerial part poultice with olive oil applied on hematomas.	[89]
Oil	Arthritis	*Rosmarinus officinalis* L. crushed leaves put in infusion in olive oil to treat arthritis.	[89]
Oil	Rheumatism	By massaging warm oil.	[78]
Oil	Rheumatism	Topical use of crushed leaves of *Brassica oleracea* L. s.l. with olive oil.	[78]
Oil	Anti-rheumatic	An olive oil macerate of *Capsicum annuum* L. fruits is used for anti-rheumatic massages.	[93]
**Diseases of the genitourinary system**			
Leaves	Kidney stones	A glass drunk early morning on an empty stomach of a decoction made from three cloves (‘strusci’) of garlic and seven olive leaves. Alternatively, a decoction of merely the leaves (“it’s very bitter, but does you good”).	[86]
Oil	Diuretic	*Borago officinalis* aerial parts decoction mixed with olive oil.	[130]
Leaves	Urinary trait diseases		[110]
Oil	Menstrual pains	Compress of oil and chamomile applied to the belly against menstrual pains.	[111]
**Pregnancy, childbirth and puerperium**			
Leaves	Abortive	Decoction.	[102]
**Injury, poisonings and certain other consequences of external causes**			
Oil (warm)	Sprains	Red-hot coal-shovel or tongs were immersed in the oil to prepare “olio ferrato” with fried oil. This was applied with cotton wool and kept in place with a woolen scarf.	[86]
Branches	Wounds/plagues	The decoction of boiled tender branches is externally used and has astringent and antiseptic action when washing sores.	[82]
Leaves	Wounds	Topical use of the decoction.	[96]
Oil	Wounds	Gallis of *Quercus cerris* L/*Quercus pubescens* Willd. powder (mixed in olive oil).	[88]
Oil	Wounds/antiseptic	Raw leaves and fruits of *Rubus ulmifolius* Schott mixed in olive oil.	[88]
Oil	Wounds	Topical use of oil.	[78]
Oil	Wounds	Topical use.	[102]
Oil	Wounds	An ointment made with the bark of *Ulmus minor* Mill. and olive oil is used to treat/promote wound healing.	[81]
Oil	Anti-edematous/anti-inflammatory	Olive oil is employed, together with wine and flour for preparation of a plaster with *Triticum aestivum*. It is applied locally on hematomas, oedemas and sprained joints.	[87]
Oil	Skin abscesses	Membranes of the bulbs of *Allium cepa* frying with olive oil to heal purulent skin abscesses (caused by thorns).	[119]
Leaves	Wounds		[110]
Oil	Wounds	Applications.	[111]
Oil	Contusions	Topical use of crushed leaves of *Brassica oleracea* L. s.l. with olive oil.	[78]
Oil	Hematomas	*Parietaria judaica* L. aerial part poultice with olive oil applied on hematomas.	[89]
Oil	Wasp/bee stings	Minced fresh leaves of *Petroselinum crispum* (Miller). A.W. Hill in olive oil are rubbed on the skin in case of wasp or bee stings.	[84]
Oil	Earache/extraneous bodies	Some warm drops of oil soothes earache and enables extraneous bodies to be removed from the ear, such as small insects. After the instillation the oil is allowed to seep out bringing the insect with it. This manipulation brings relief and eliminates the sensation of having blocked ears.	[84]
Oil	Sprains and dislocations	The aching part is massaged with warm oil.	[115]
Oil	Venomous stings and bites	Oil in which a live gecko (‘Tattaruledda’) is soaked and applied locally.	[115]
Oil	Dislocations	Topical use.	[102]
Oil	Sprains pain	*Parietaria judaica* L. aerial part poultice with olive oil applied on hematomas.	[89]
Oil	To remove foreign objects	To facilitate the removal of thorns from the skin.	[89]
Oil	Sprain pains	Topical use of crushed leaves of *Brassica oleracea* L. s.l. with olive oil.	[78]
Oil	Sprain pains	By massaging warm oil with leaves of *Ruta chalepensis*.	[78]
Oil	Bruises	*Arnica montana* macerate in olive oil, externally applied.	[128]
Oil	Anti-inflammatory	A poultice of fresh leaves of *Vincetoxicum hirundinaria* Medik in olive oil with beeswax is claimed to be an anti-inflammatory in case of trauma.	[93]
Oil	Bruises	*Parietaria judaica* L. aerial part poultice with olive oil applied on hematomas.	[89]
**Symptoms not elsewhere classified**			
Leaves	Fever	Olive leaves are a well-known febrifuge.	[82]
Oil	High temperature	Rubbed onto the chest (high temperature) and ears.	[86]
Leaves	Fever	Decoction as febrifuge.	[105]
Oil (extract)	Anti-inflammatory	Used as anti-inflammatory.	[106]
Oil	Analgesic/Anti-inflammatory	Sambucus nigra dried flowers warmed up in olive oil and massaged on painful or inflamed body parts, also applicable to animals.	[90]
Oil	Anti-inflammatory	As a treatment for parotitis warm boiled leaves of *Cynoglossum creticum* mashed together with olive oil, are applied locally then wrapped up in bandages.	[87]
Oil	Inflammation	Olive oil used against inflammation.	[111]
Oil	Vitamin deficiency	Olive oil used against vitamin deficiency.	[111]
Oil	As analgesic	The fresh leaves of *Ruta graveolens* L. are macerated in olive oil, then filtered and rubbed in as a local analgesic.	[84]
Leaves	Sedative	Infusion as sedative.	[131]
Leaves	Water retention	Infusion.	[116]

## Data Availability

The data generated during the current study are available within the article.

## Supplementary Materials

The following supporting information can be downloaded at https://www.mdpi.com/article/10.3390/plants15142221/s1, Table S1: The role of olive oil as vehicle of improbable substances in the bizarre remedies against the most diverse pathologies reported by Pliny the Elder in Book XXX of *Naturalis Historia*.
