# Supplementary material for: The Ethnobotanical Heritage of Olea europaea L. in Italy: Continuity of a Millenary Mediterranean Tradition
Source: Plants (Basel). 2026 Jul 21;15(14):2221. doi: 10.3390/plants15142221 (PMC13415444; doi:10.3390/plants15142221)
Supplement: Supplementary file 1 [file plants-15-02221-s001.zip › plants-4416196-supplementary.pdf]

Table S1. The role of olive oil as vehicle of improbable substances in the bizarre remedies against the most diverse pathologies reported by Pliny the Elder in Book XXX of *Naturalis Historia*

**Chapter**	**Administration**	**Pathology**
VIII	earthworms cooked in oil and infused in the ears	tooth-ache
	snakeskin after the spring moult/ sparrow dung, mixed with hot oil and infused in the ears	gum-ache
X	a balm made of hedgehog ash and oil	impetigo
XII	pigeon dung mixed with wine and oil	quinsy
	snakes' ash/fat mixed with oil	scrofula
XIV	skinned mice-better the African ones- boiled in oil with salt and eaten	lung diseases
XIX	oil mixed with roasted skin from a hen abstaining from cereals	abdominal pain in celiacs
XXIII	embers of burnt mice/ dormice/ earthworms/pigeons' dung mixed with oil/old oil, earthworms in oil, tarantula head with *Vitix* leaves chopped in oil, hen dung with saltpetre and oil	ulcers/warts/calluses on feet
XXIV	a dragon's intestines and oil -plus wine- decoction	agitation due to night gods and fauns
XXIX	an ointment made of oil mixed with burnt combs, nails and ears of chickens, when the sun passes through Gemini, of oil mixed with burnt spurs and wattles if the moon passes through Gemini	fevers
XXXV	dormouse with oil and pigeon dung spread with oil	sunburns
XXXVII	a vulva cooked in oil	whitlows and excrescencies on fingers
XXXIX	an equal amount of burnt woodworms and anise with oil, barn owl cooked in oil	ulcers
XL	a dog's brain smeared upon a cloth covered with wool and oil	broken bones
XLI	the white part of hems' dung mixed with old oil kept in a horn box	morphew
XLIII	ashes of a burnt hedgehog applied with oil	preventing abortion
XLVI	boneless snake boiled in 3 *heminae* -0.81 l- of oil	growth of unwanted hair
	hoaryness	earthworms' ashes mixed with oil
XLIX	drinking pigeon dung mixed with oil and wine	antaphrodisiac
